# Facilitating early diagnosis of chronic thromboembolic pulmonary hypertension with dynamic chest radiography: Protocol for a multicenter, assessor-blinded, case-wise randomized superiority reader study (FIND-DCR)

**DOI:** 10.1371/journal.pone.0350858

**Published:** 2026-06-11

**Authors:** Kazuya Hosokawa, Yuzo Yamasaki, Junji Kishimoto, Tomomi Nagayama, Shiro Adachi, Shinji Naganawa, Takumi Inami, Kenichi Yokoyama, Satoshi Yasuda, Kei Takase, Kayoko Kubota, Takashi Yoshiura, Toshihiko Sugiura, Takashi Uno, Masaharu Kataoka, Takatoshi Aoki, Soichiro Usui, Satoshi Kobayashi, Yu Taniguchi, Takamichi Murakami, Yuichi Tamura, Yoshihiro Fukumoto, Kousei Ishigami, Kohtaro Abe

**Affiliations:** 1 Department of Cardiovascular Medicine, Graduate School of Medical Sciences, Kyushu University, Fukuoka, Japan; 2 Center for Clinical and Translational Research, Kyushu University Hospital, Fukuoka, Japan; 3 Department of Clinical Radiology, Graduate School of Medical Sciences, Kyushu University, Fukuoka, Japan; 4 Department of Cardiology, Nagoya University Hospital, Nagoya, Japan; 5 Department of Radiology, Nagoya University Graduate School of Medicine, Nagoya, Japan; 6 Department of Cardiovascular Medicine, Kyorin University School of Medicine, Tokyo, Japan; 7 Department of Radiology, School of Medicine, Kyorin University, Tokyo, Japan; 8 Department of Cardiovascular Medicine, Tohoku University Graduate School of Medicine, Sendai, Japan; 9 Department of Diagnostic Radiology, Tohoku University Graduate School of Medicine, Sendai, Japan; 10 Department of Cardiovascular Medicine, Kagoshima University Graduate School of Medical and Dental Sciences, Kagoshima, Japan; 11 Department of Radiology, Kagoshima University Graduate School of Medical and Dental Sciences, Kagoshima, Japan; 12 Department of Respirology, Chiba University Graduate School of Medicine, Chiba, Japan; 13 Diagnostic Radiology and Radiation Oncology, Graduate School of Medicine, Chiba University, Chiba, Japan; 14 The Second Department of Internal Medicine, University of Occupational and Environmental Health, Kitakyushu, Japan; 15 Department of Radiology, University of Occupational and Environmental Health, Kitakyushu, Japan; 16 Department of Cardiovascular Medicine, Graduate School of Medical Sciences, Kanazawa University, Kanazawa, Japan; 17 Department of Radiology, Graduate School of Medical Sciences, Kanazawa University, Kanazawa, Japan; 18 Department of Cardiovascular Medicine, Graduate School of Medicine, Kobe University, Kobe, Japan; 19 Department of Radiology, Kobe University Graduate School of Medicine, Kobe, Japan; 20 Pulmonary Hypertension Center, International University of Health and Welfare Mita Hospital, Tokyo, Japan; 21 Division of Cardiovascular Medicine, Department of Internal Medicine, Kurume University School of Medicine, Kurume, Japan; Osaka University Graduate School of Medicine, JAPAN

## Abstract

**Introduction:**

Chronic thromboembolic pulmonary hypertension (CTEPH) is a treatable cause of pulmonary hypertension but remains under-recognized and is often diagnosed with delay. Limited access to lung ventilation-perfusion (V/Q) scintigraphy, especially outside tertiary centers, is one contributor. Dynamic chest radiography (DCR), with a pulmonary circulation analysis program, can provide a rapid, non-invasive, and widely deployable assessment of pulmonary perfusion. We describe the protocol of a multicenter reader study testing whether adding DCR-based analysis to standard initial work-up improves diagnostic accuracy for CTEPH among patients with echocardiographically suspected pulmonary hypertension.

**Methods and analysis:**

This investigator-initiated, multicenter, assessor-blinded, case-wise randomized superiority reader study compares standard initial work-up (blood tests, chest X-ray, ECG, pulmonary function tests, and transthoracic echocardiography per guidelines) with standard work-up plus DCR-based pulmonary circulation analysis. The primary endpoint is diagnostic accuracy for discrimination between CTEPH and non-CTEPH in the intention-to-treat set. The final diagnosis of CTEPH versus non-CTEPH will be defined as the reference standard according to the Japanese and European guidelines for pulmonary hypertension. Secondary endpoints include sensitivity, specificity, positive and negative predictive values; agreement with V/Q scintigraphy regarding regional perfusion defects using κ statistics; and STARD-conformant academic performance evaluation of DCR-based pulmonary circulation analysis and lung perfusion scintigraphy in relation to the site-level final diagnosis of CTEPH versus non-CTEPH in a full analysis set. Safety endpoints include adverse events during DCR acquisition and device malfunctions. The target sample size is 108 cases with 1:1 allocation. Recruitment started on 30/07/2025 and is expected to continue until 28/02/2027, with overall study completion planned for 31/05/2027.

**Discussion:**

This multicenter reader study addresses a key limitation of current CTEPH diagnostic pathways—reliance on V/Q scintigraphy, which may be delayed or unavailable outside tertiary centers—by evaluating whether DCR-based pulmonary circulation analysis can improve early discrimination of CTEPH and support timely referral.

**Trial registration number:**

Japan Registry of Clinical Trials (jRCT), jRCT2072250027.

## Introduction

**Fig 1:**
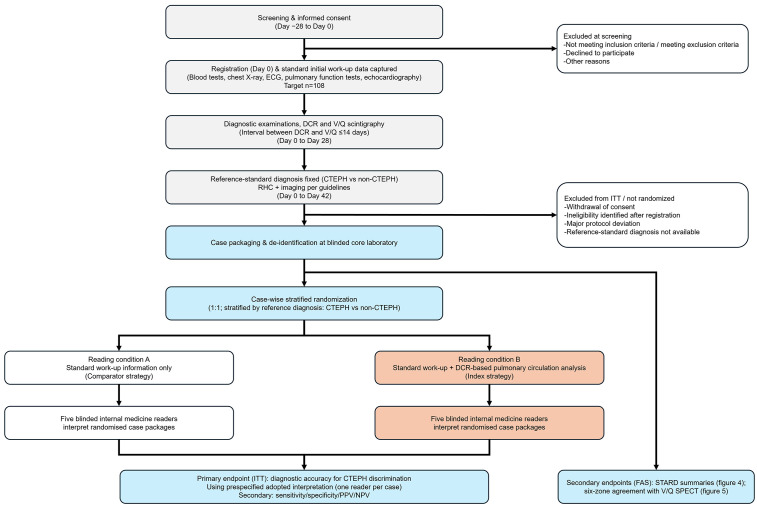
SPIRIT flow diagram of the FIND-DCR study. After screening and informed consent, participants are registered and undergo standard initial work-up. Dynamic chest radiography (DCR) and V/Q scintigraphy are performed, and the reference diagnosis (CTEPH vs non-CTEPH) is fixed using right heart catheterization and guideline-recommended imaging. De-identified case packages are randomized 1:1 to reading condition A (standard work-up only) or B (standard work-up plus DCR outputs) and interpreted by five blinded readers; the primary endpoint is diagnostic accuracy. CTEPH, chronic thromboembolic pulmonary hypertension; DCR, dynamic chest radiography; ECG, electrocardiogram; ITT, intention-to-treat; NPV, negative predictive value; PPV, positive predictive value; RHC, right heart catheterization; SPECT, single-photon emission computed tomography; STARD, Standards for Reporting Diagnostic Accuracy Studies; V/Q, ventilation-perfusion.

Chronic thromboembolic pulmonary hypertension (CTEPH) is a treatable cause of pulmonary hypertension (PH) through pulmonary endarterectomy and balloon pulmonary angioplasty [1,2]. However, CTEPH remains underdiagnosed, and reported delays from symptom onset to diagnosis frequently exceed one year in routine practice [3,4]. Limited availability and underuse of lung ventilation-perfusion (V/Q) scintigraphy, particularly outside tertiary centers with nuclear medicine facilities, contribute to diagnostic care gaps, delayed diagnostic pathways, and missed opportunities for timely referral [5,6]. The overall study design is outlined in Fig 1. Although dynamic digital radiography is not yet as widely disseminated as conventional static radiography, its clinical availability has been gradually increasing since its commercial introduction. Because it can be implemented in a general radiographic imaging environment without contrast media, radionuclides, or dedicated nuclear medicine infrastructure, DCR may offer a practical advantage in settings where access to V/Q scintigraphy is limited. This issue is particularly relevant given the logistical and economic constraints associated with scintigraphy, including radiotracer availability, equipment costs, and the sustainability of isotope supply. If its clinical utility is established, DCR-based pulmonary circulation analysis could become a more accessible tool for early pulmonary circulation assessment in both expert and non-expert settings.

**Fig 2:**
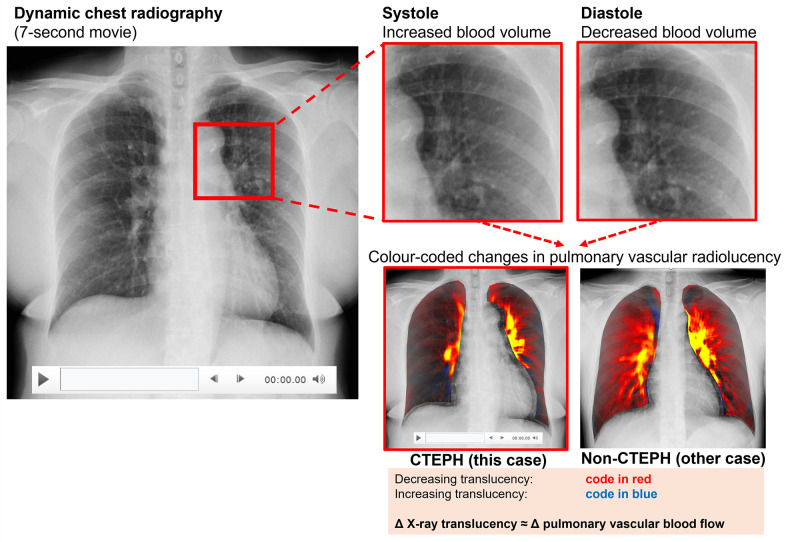
Mechanism of pulmonary circulation analysis based on dynamic chest radiography. Dynamic chest radiography captures cyclic changes in pulmonary blood volume using pulsed X-ray sequences. The dedicated pulmonary circulation analysis program overlays perfusion-surrogate color maps to visualize regional pulmonary blood flow without contrast media. A sample of the DCR-based pulmonary circulation movie is provided in S1 File.

Dynamic chest radiography (DCR) leverages pulsed X-ray sequences and flat-panel detectors to visualize cardio-cyclic changes in pulmonary vascular shadow intensity. Using a dedicated pulmonary circulation analysis program, DCR can generate color-coded perfusion surrogates and summary maps of regional blood flow without intravenous contrast administration (Fig 2). Preliminary single-center retrospective work has shown high agreement between DCR-derived images and V/Q scintigraphy in detecting CTEPH [7]. Table 1 compares key features of diagnostic tools for pulmonary circulation-V/Q scintigraphy, DCR-based pulmonary circulation analysis, and conventional static chest radiography. DCR-based pulmonary circulation analysis is a rapid, simple, non-invasive test with ultra-low radiation exposure that can visualize regional pulmonary perfusion surrogates [8].

**Table 1:** Comparison of features between V/Q scan, dynamic chest radiography plus pulmonary circulation analysis program, and chest radiography.

	Lung ventilation–perfusion (V/Q) scintigraphy	Dynamic chest radiography/ pulmonary circulation analysis program	Chest radiography
**Assessment focus**	Defects in pulmonary arterial perfusion	Defects in pulmonary vascular perfusion	Interruption or abnormal caliber of pulmonary vascular markings; presence or absence of pulmonary infarction
**Diagnostic performance for CTEPH**	Very high	High (according to previous study)	Low
**Examination time (room stay)**	≈30 min	<5 min	<5 min
**Invasiveness**	Intravenous injection of radiotracer	None	None
**Radiation exposure**	1.2–2.0 mSv	0.1–0.4 mSv	0.1 mSv
**Resource/economic burden**	High	Low	Low
**Note**	Recommended in guidelines	Novel modality; limited prospective evidence to date	Primarily for initial work-up

CTEPH, chronic thromboembolic pulmonary hypertension.

To date, there is no widely available tool for assessing pulmonary circulation at the time of initial clinical evaluation, particularly in non-expert or community settings. The standard initial work-up, including blood tests, chest radiography, ECG, pulmonary function tests, and echocardiography, provides solely indirect and often non-specific evidence of pulmonary hypertension and its potential causes. In contrast, DCR-based pulmonary circulation analysis provides additional dynamic regional perfusion-surrogate information derived from cardiac cycle-related changes in X-ray translucency during a short breath-hold, without the need for contrast media or radionuclides. FIND-DCR is the first prospective comparative study to test, in a controlled multicenter setting, whether adding DCR-based pulmonary circulation analysis to the standard initial work-up improves the initial discrimination between CTEPH and non-CTEPH in patients with echocardiographically suspected PH. If successful, this strategy could help prioritize referral to expert PH centers for resource-intensive but definitive investigations such as V/Q scintigraphy.

## Objectives and hypotheses

The primary objective, defined in prior consultation with the Pharmaceuticals and Medical Devices Agency (PMDA, Japan’s regulatory authority for drugs, medical devices, and regenerative medical products), is to determine whether standard initial work-up plus DCR-based pulmonary circulation analysis yields superior diagnostic accuracy for the initial discrimination between CTEPH and non-CTEPH compared with standard work-up alone. In addition, this study will evaluate the diagnostic performance of DCR-based pulmonary circulation analysis versus lung V/Q SPECT for discriminating CTEPH from non-CTEPH and will report diagnostic accuracy measures in accordance with STARD 2015.

We hypothesize that adding DCR-based analysis to standard work-up will be superior in terms of diagnostic accuracy for initial CTEPH discrimination compared with standard work-up alone.

## Methods and analysis

### Study design and setting

The FIND-DCR study is an investigator-initiated, multicenter, assessor-blinded, case-wise randomized superiority reader study that compares two diagnostic strategies for the initial discrimination of CTEPH versus non-CTEPH in adults with echocardiographically suspected PH.

For each enrolled participant, a de-identified case package is created after completion of the planned diagnostic work-up and determination of the reference-standard diagnosis (CTEPH vs non-CTEPH) by the site investigator. Cases are stratified by the definitive diagnosis (CTEPH vs non-CTEPH) and then randomized in a 1:1 ratio to be interpreted either (a) with standard initial work-up information alone (comparator strategy) or (b) with standard initial work-up information plus DCR-based pulmonary circulation analysis outputs (index strategy). Within the blinded core laboratory, five internal medicine physicians independently interpret each case in its allocated strategy. For the prespecified confirmatory primary endpoint, a single case-level classification is derived using a prespecified correspondence table that uniquely selects one reader’s judgement for each case.

Readers are blinded to the reference-standard diagnosis and to subsequent clinical outcomes beyond the initial work-up. De-identified imaging and clinical data (e.g., age, sex, blood tests, pulmonary function tests, 12-lead ECG, static chest X-ray, transthoracic echocardiography, and dynamic chest radiography) are transferred to a blinded central core laboratory, where case packaging, image processing and reader adjudication are performed according to prespecified standard operating procedures. Table 2 summarizes the study, Fig 1 shows the study flow, and Table 3 presents the visit and assessment schedule.

**Table 2:** Summary of study objectives associated with outcomes.

Objective	To verify that adding dynamic chest radiographic imaging and the pulmonary circulation analysis program to the initial work-up improves the accuracy of differentiating CTEPH from non-CTEPH.
**Investigational device**	Pulmonary circulation analysis program using dynamic chest radiography.
**Control group**	Initial work-up group (including blood tests, standard chest radiography, 12-lead electrocardiogram, pulmonary function tests, and transthoracic echocardiography).
**Test group**	Examination group in which the initial work-up is supplemented with pulmonary circulation analysis using the investigational device (device-added group).
**Target disease**	Patients in whom pulmonary hypertension is observed on transthoracic echocardiography and in whom chronic thromboembolic pulmonary hypertension (CTEPH) is suspected.
**Study design**	Multicenter, assessor-blinded, case-wise randomized, comparative diagnostic performance, superiority image-reading study.
**Endpoints**	**Primary endpoint**
	Using the standard initial work-up alone as the control examination, the diagnostic accuracy of the initial differentiation between CTEPH and non-CTEPH in the examination group in which dynamic chest radiographic imaging and the pulmonary circulation analysis program are added to the initial work-up (device-added group).*
	**Secondary endpoints**
	Using the standard initial work-up alone as the control examination, the sensitivity, specificity, positive predictive value, and negative predictive value of the initial differentiation between CTEPH and non-CTEPH in the device-added group. *
	For the diagnosis of CTEPH versus non-CTEPH, the sensitivity, specificity, accuracy, positive predictive value, and negative predictive value of lung perfusion scintigraphy and of dynamic chest radiographic imaging/ the pulmonary circulation analysis program. **
	Agreement (κ coefficient) between dynamic chest radiographic imaging/ the pulmonary circulation analysis program and lung perfusion scintigraphy regarding the presence of regional perfusion defects in each of the six lung regions. *
**Sample size**	108 patients (1:1 allocation).
**Study period**	June 2025 – May 2027.

CTEPH, chronic thromboembolic pulmonary hypertension.

* According to PMDA agreements

** According to STARD guidelines

**Table 3:** Schedule of enrolment, diagnostic procedures, and image interpretation in the FIND-DCR study.

	Informed consent	Eligibility screening	Registration	Diagnostic tests			Image processing at blinded core laboratory
Visit window	Day-28~0		Index date (Day 0)	Day 0~28	Day -28~42	Day 0~42	
**Informed consent**	✓						
**Eligibility**			✓				
**Registration**			✓				
**Past medical history, comorbidities**		✓					
**Height, weight**		✓					
**WHO functional class**		✓					
**Blood test**		✓					
**12-lead ECG**		✓					
**Pulmonary function test**		✓					
**Echocardiogram**		✓					
**Chest X-ray**		✓					
**Dynamic chest radiography**				✓			
**VQ scan, SPECT**				✓			
**Right heart catheterization**					✓		
**Definitive diagnosis of CTEPH/non-CTEPH**						✓	
**Adverse event**				✓			
**Device malfunction**				✓			✓

After enrollment, standard initial work-up examinations are performed, followed by dynamic chest radiography and lung ventilation-perfusion scintigraphy within 28 days, with an interval of no more than 14 days between the two examinations. The reference-standard diagnosis (CTEPH vs non-CTEPH) is determined by the site investigator within 42 days after registration. Case-wise randomization and blinded image interpretation at the central core laboratory are then performed. Safety assessments include adverse events related to dynamic chest radiography and device malfunctions. CTEPH, chronic thromboembolic pulmonary hypertension; ECG, electrocardiogram.

Participating centers include Japanese tertiary and referral hospitals with expertise in PH and access to lung ventilation-perfusion (V/Q) scintigraphy and right heart catheterization (RHC). Recruitment started on 30/07/2025 and is expected to continue until 28/02/2027 (recruitment period: 30/07/2025–28/02/2027), and overall study completion is planned for 31/05/2027.

## Participants and eligibility criteria

### Inclusion criteria

- Age ≥ 18 years at the time of consent.
- High echocardiographic probability of PH according to major guidance (Japanese and European guidelines) [2,9] and scheduled to undergo lung V/Q scintigraphy and RHC for definitive diagnosis of CTEPH versus non-CTEPH.
- Ability to understand the study information and provision of written informed consent.

### Key exclusion criteria

- Severe obstructive lung disease (FEV₁ < 60% predicted) or severe restrictive lung disease (total lung capacity <60% predicted) at screening, or PH that can be explained by Group 3 lung disease as judged by the investigator.
- Severe aortic or mitral valve disease or left ventricular ejection fraction <40% on transthoracic echocardiography at screening, or PH that can be explained by Group 2 left heart disease as judged by the investigator.
- Poorly controlled tachyarrhythmia (resting heart rate >100 beats per minute).
- Acute symptomatic pulmonary embolism on imaging within 3 months prior to enrollment.
- Congenital or acquired abnormalities of pulmonary vascular anatomy.
- History of lung resection or chest radiotherapy.
- Active thoracic malignancy.
- History of vasculitis.
- Presence of devices in the chest (such as pacemakers or implantable cardioverter-defibrillators) in which pulsed X-ray exposure is contraindicated because of potential device malfunction.
- Pregnant women.
- Any other condition judged by the investigator to make participation inappropriate.

### Index and comparator diagnostic strategies

The comparator strategy (standard initial work-up) comprises:

- Blood tests; full blood count; biochemistry (total protein, albumin, urea nitrogen, creatinine, uric acid, total bilirubin, AST, ALT, LDH, C-reactive protein, sodium, potassium, chloride, estimated glomerular filtration rate); D-dimer; NT-proBNP,
- Static chest X-ray,
- 12-lead ECG,
- Pulmonary function tests; total lung capacity, vital capacity, FEV₁ and FEV₁/FVC, and
- Transthoracic echocardiography.

The index strategy (standard work-up plus DCR) adds DCR acquisition and pulmonary circulation analysis using a dedicated program (investigational device). The program generates dynamic sequences and summary perfusion-surrogate images, which are used by readers as part of the diagnostic strategy. For initial discrimination in the index strategy, if a regional perfusion defect is identified on the pulmonary circulation analysis images, the case will be classified as CTEPH.

### Dynamic chest radiography acquisition

**Fig 3:**
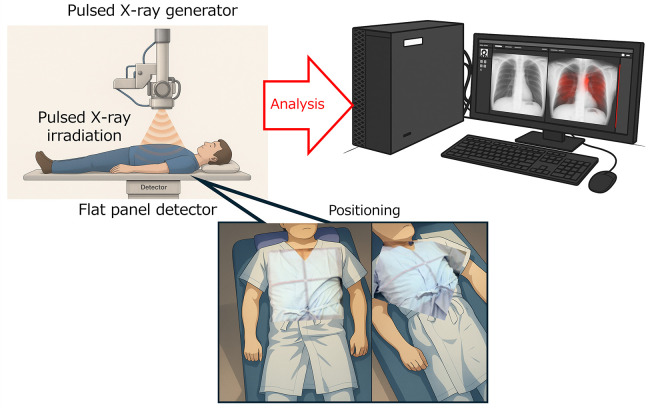
Overview of dynamic chest radiography acquisition and pulmonary circulation analysis. Dynamic chest radiography is performed using a pulsed X-ray generator and a flat-panel detector while the patient holds their breath in the supine anteroposterior position (or upright posteroanterior position, where applicable). Sequential X-ray images acquired during pulsed X-ray irradiation are transferred to a dedicated workstation, where the pulmonary circulation analysis program processes the image series to generate dynamic and summary perfusion-surrogate images. Representative examples of patient positioning for dynamic chest radiography are shown.

DCR is acquired using a pulsed X-ray sequence with a flat-panel detector. After enrollment, DCR and lung V/Q scintigraphy are performed within 28 days after registration, with an interval between the two examinations of ≤14 days. Acquisition is performed in either an anteroposterior (supine; recommended) or posteroanterior (standing) projection, with coached breath-hold for 6–10 seconds (typically 7 seconds). The standard acquisition frame rate is approximately fifteen frames per second. An overview of dynamic chest radiography acquisition and pulmonary circulation analysis is shown in Fig 3.

Image quality is assessed immediately after acquisition. Adequate DCR requires (i) breath-hold of at least 6 seconds, (ii) full coverage of both lung fields, (iii) no severe motion artifact and (iv) no major frame dropouts. When the initial acquisition is inadequate and a repeat is feasible, acquisition may be repeated as needed according to the prespecified imaging criteria. Acquisition posture, frame rate, breath-hold duration, dose-area product (radiation exposure), number of acquisition attempts and DCR room time are recorded prospectively.

### Pulmonary circulation analysis workflow

DCR sequences are processed at the blinded central core laboratory using the dedicated pulmonary circulation analysis program. The program performs motion correction and quantifies cardio-cyclic changes in pulmonary vascular shadow intensity to generate color-coded perfusion-surrogate images and summary maps of regional pulmonary blood flow (Fig 2). All processing steps follow a prespecified workflow and are performed by trained operators who are blinded to the reference-standard diagnosis.

**Fig 4:**
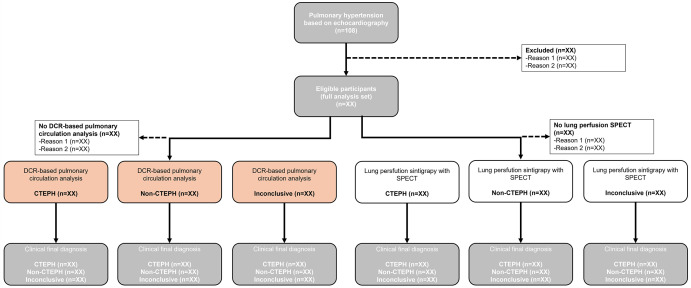
Perfusion-defect detection performance of dynamic chest radiography versus lung perfusion SPECT. Agreement between dynamic chest radiography-based pulmonary circulation analysis and lung perfusion SPECT for detecting regional pulmonary perfusion defects across six predefined lung zones, quantified using κ statistics.

The derived outputs provided to DCR readers include the DCR cine sequence and the program-generated perfusion-surrogate images. For regional analyses performed in a predefined analysis set irrespective of the primary randomization, the lungs are divided into six predefined zones (right/left upper, middle and lower regions; Fig 4), and readers record the presence or absence of embolic-type perfusion defects in each zone. Static chest radiography (e.g., conventional chest X-ray) and V/Q mismatch are not considered in this six-zone assessment.

### Reader training, calibration, and image interpretation

Separate groups of readers will interpret the DCR-based pulmonary circulation images and the perfusion scintigraphy studies. DCR will be read by internal medicine physicians who have not worked full-time at a CTEPH expert center certified by the Japanese Pulmonary Circulation and Pulmonary Hypertension Society within the past 3 years. Perfusion scintigraphy will be interpreted by nuclear medicine physicians (or radiologists with expertise in nuclear medicine) who currently work, or have worked within the past 3 years, at a CTEPH expert center. There will be no overlap in personnel between the two reader groups.

Before formal image interpretation, DCR readers will complete a calibration set of 20 representative cases balanced for CTEPH/non-CTEPH status and image quality and will participate in a prespecified training workshop to standardize interpretation criteria, the six-zone regional labeling scheme, and the handling of image artifacts. Reader eligibility will be confirmed by completion of a prespecified qualification assessment based on the training cases. This training-based approach is supported by prior work on DCR in CTEPH, in which readers were trained using representative CTEPH and non-CTEPH cases before image interpretation and substantial inter-reader agreement for DCR was achieved (κ = 0.71) [7].

During the formal reading phase, five DCR readers will interpret all randomized case packages independently in their allocated strategy using a standardized case report form. Each case will be interpreted only once in its allocated condition, and readers will not review the same case both with and without DCR outputs. During the reading phase, no feedback regarding diagnostic correctness will be provided to the readers. For the primary endpoint, each reader will provide (i) a binary judgement (CTEPH vs non-CTEPH) and (ii) six-zone regional perfusion defect assessments. Non-evaluable interpretations due to non-diagnostic image quality are permitted and will be handled as prespecified.

For the confirmatory primary endpoint, a prespecified correspondence table will be used to derive an adopted interpretation such that a single reader’s judgement is uniquely selected for each case, rather than by simple majority vote, while balancing reader experience. For secondary endpoints requiring pooled reader decisions, determinations will be made by majority vote within the relevant reader panel, as prespecified. Perfusion scintigraphy images for the six-zone assessment will be interpreted independently by a separate panel of three nuclear medicine physicians, blinded to DCR outputs and to the reference-standard diagnosis. Using SPECT/CT (or SPECT when CT is unavailable) to define the cranio-caudal extent of the lungs, each lung will be divided into three equal zones (upper, middle, and lower), resulting in six zones. For each zone, readers will record ‘present’ when an embolic-type perfusion abnormality is judged to be present and ‘absent’ when it is judged not to be present; the final zone-level result will be determined by majority vote. Ventilation scintigraphy is not used for this six-zone assessment; therefore, V/Q mismatch is not considered in the regional agreement analyses.

### Reference standard and clinical verification

The reference-standard diagnosis (CTEPH vs non-CTEPH) will be determined by the principal investigator or subinvestigator at each participating site within 42 days after registration. To diagnose CTEPH, concordant findings must be present on V/Q scintigraphy, organized thrombi must be identified within the pulmonary arteries on at least one modality (CT pulmonary angiography or catheter-based pulmonary angiography), and pulmonary hypertension must be confirmed by right heart catheterization (hemodynamic criteria: mean pulmonary arterial pressure >20 mmHg and pulmonary capillary wedge pressure ≤15 mmHg). Non-CTEPH will include cases with an alternative definitive diagnosis or no pulmonary hypertension on right heart catheterization. An inconclusive diagnosis due to insufficient data, as judged by the investigator, may be classified as non-evaluable for this study. The site-level final diagnosis of CTEPH versus non-CTEPH serves as the reference standard for the planned performance evaluations in the blinded core laboratory.

### Ethics statement

The study will be conducted in accordance with the Declaration of Helsinki, the International Council for Harmonization Good Clinical Practice guidelines, and applicable national regulations. Institutional review board approval was first obtained at the coordinating institution (Institutional Review Board of Kyushu University Hospital; ID 2025302, 30/07/2025) and was subsequently obtained at each participating hospital, including Tohoku University (ID 253007, 22/12/2025), Kyorin University (ID 2513, 20/11/2025), Chiba University (ID K2025002, 17/11/2025), Nagoya University (ID 372005, 22/12/2025), Kanazawa University (ID 9045, 08/10/2025), Kobe University (ID 250025A, 05/11/2025), the University of Occupational and Environmental Health (ID 10583, 22/10/2025), and Kagoshima University (ID 25012, 22/10/2025). Written informed consent will be obtained from all participants prior to enrollment.

### Outcomes

The primary endpoint (case-level diagnostic accuracy for the initial discrimination between CTEPH and non-CTEPH) has been defined as a prespecified confirmatory outcome in consultation with PMDA, the Japanese regulatory authority for drugs, medical devices, and regenerative medical products. From an academic perspective, diagnostic performance will be analyzed and reported in accordance with the STARD 2015 guideline [10].

### Primary endpoint (ITT set)

- Diagnostic accuracy for initial discrimination between CTEPH and non-CTEPH, comparing standard work-up plus DCR versus standard work-up alone.

### Secondary endpoints

**Fig 5:**
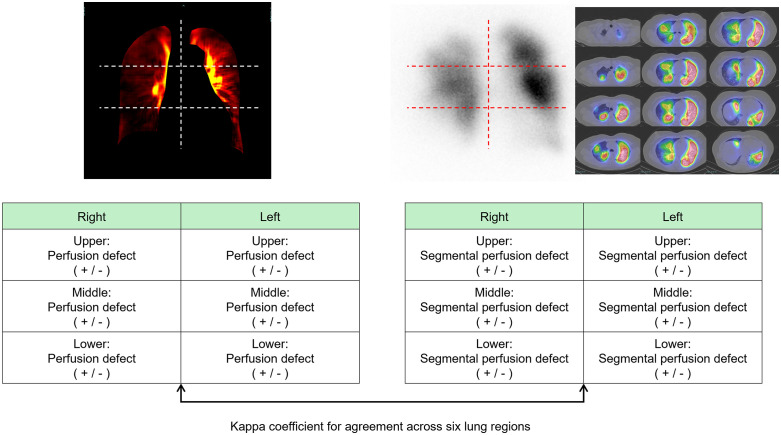
Study flow for the secondary outcome (STARD-based evaluation). Analysis set for STARD-conformant diagnostic accuracy of DCR-based pulmonary circulation analysis and lung perfusion scintigraphy in relation to the site-level final diagnosis of CTEPH versus non-CTEPH, including evaluation of sensitivity, specificity, accuracy, PPV and NPV. CTEPH, chronic thromboembolic pulmonary hypertension; DCR, dynamic chest radiography.

- Sensitivity, specificity, positive predictive value, and negative predictive value for CTEPH versus non-CTEPH discrimination for each diagnostic strategy (ITT set).
- STARD-based academic performance evaluation (sensitivity, specificity, accuracy, positive predictive value, and negative predictive value) of pulmonary blood flow defect detection by DCR-based pulmonary circulation analysis and lung perfusion scintigraphy in relation to the site-level final diagnosis of CTEPH versus non-CTEPH (full-analysis set) (Fig 5).
- Agreement (kappa (κ) coefficient) between DCR-based analysis and V/Q scintigraphy regarding the presence of regional perfusion defects in each of six lung regions (full-analysis set) (Fig 4).

### Safety endpoints

- Adverse events occurring during or immediately after DCR acquisition.
- Device malfunctions during the study.
- Procedural metrics: DCR room time, dose-area product (radiation exposure), re-take rate, and reasons for image exclusion.

### Sample size

The sample size calculation was based on diagnostic accuracy for discriminating CTEPH from non-CTEPH. The 2022 ESC/ERS pulmonary hypertension guidelines [2] and a retrospective study from Hammersmith Hospital [11] indicate that lung ventilation-perfusion (V/Q) scintigraphy has high sensitivity and specificity for CTEPH with reported sensitivity and specificity of 90–100% and 94–100%, respectively. Perfusion-only SPECT/CT demonstrated high diagnostic performance for chronic thromboembolic pulmonary hypertension in comparison with ventilation-perfusion planar, SPECT, and SPECT/CT imaging [12]. In a retrospective study at Kyushu University Hospital, both V/Q scintigraphy and the dynamic chest radiography (DCR)-based pulmonary circulation analysis program showed high accuracy (92% and 94%, respectively) and strong agreement (κ = 0.79) [7], whereas an initial work-up consisting of chest radiography, blood tests (including BNP), ECG and pulmonary function tests achieved a mean diagnostic accuracy of 70.5% for CTEPH versus non-CTEPH (not published).

Based on these data, we assumed an expected diagnostic accuracy of 70.5% in the standard initial work-up group and 92% in the device-added group. With 1:1 allocation, a two-sided significance level of α = 0.05 and power (1-β) = 0.80, a χ² test for two independent proportions requires 102 patients in total. Allowing for approximately 5% non-evaluable cases (e.g., missing reference-standard data or non-interpretable images), we set the target sample size at 108 patients, with fifty-four patients in each group. As the primary analysis will use a multi-reader multi-case framework, this case-based calculation is considered conservative.

### Randomization, allocation, and blinding

Case-wise randomization is performed after the reference-standard diagnosis is fixed. Using an allocation tool, cases are stratified by the definitive diagnosis (CTEPH vs non-CTEPH) to balance case mix between arms and are then allocated 1:1 to the two reading conditions. An independent statistician generates the computer-based allocation sequence, which is held at the central core laboratory. Core laboratory personnel generate the corresponding de-identified case packages (standard work-up alone vs standard work-up plus DCR outputs) and distribute them to readers via the secure reading system.

DCR readers are blinded to the reference-standard diagnosis and all results from V/Q scintigraphy and RHC. V/Q readers are blinded to DCR outputs and to the reference-standard diagnosis. Because the two reader panels are distinct and there is no overlap in personnel, cross-modality recall bias is minimized.

To mitigate learning effects, formal readings will be performed independently, in randomized case order, using standardized case report forms, and without feedback regarding diagnostic correctness during the reading phase.

### Data collection and management

Acquisition posture (supine position recommended), X-ray exposure, number of DCR re-takes, DCR room time and the time required for DCR image interpretation will be recorded prospectively. All images and clinical data are uploaded to a secure electronic data capture system that complies with relevant data protection regulations. Data queries are centrally managed, and all data handling follows sponsor-standard operating procedures. Study documents and datasets will be retained for the period required by regulations and the sponsor.

### Statistical analysis

The primary confirmatory analysis will compare diagnostic accuracy between the two strategies in the ITT set using the adopted interpretation (one prespecified reader judgement per case) as the case-level result. The difference in accuracy will be tested at a two-sided significance level of 5% (details prespecified in the statistical analysis plan) and reported with 95% confidence intervals. As a supportive analysis, multi-reader multi-case modelling will also be performed using all individual reads, treating reader and case as random effects [13].

Secondary analyses will estimate sensitivity, specificity, positive and negative predictive values and κ statistics with 95% confidence intervals in discrimination of six-zone regional perfusion defects. STARD-conformant summaries (sensitivity, specificity, accuracy, PPV and NPV) of DCR-based pulmonary circulation analysis versus lung perfusion scintigraphy in relation to the definitive diagnosis (CTEPH vs non-CTEPH) will be reported. Pre-specified subgroup analyses include sex, body mass index (≥25 vs < 25 kg/m²), pulmonary vascular resistance (>4.0 vs ≤ 4.0 Wood units), NT-proBNP (≥300 vs < 300 pg/mL), tricuspid regurgitant jet velocity (above vs below median) and cardiac rhythm (sinus vs non-sinus) and are planned as exploratory analyses. Additional supportive analyses will evaluate the influence of reader-related factors, particularly years of clinical experience, on diagnostic accuracy in DCR interpretation. No formal interim efficacy analysis or multiplicity adjustment is planned.

### Missing data, indeterminate interpretations, and indeterminate final diagnoses

Analysis sets are predefined in the protocol. Briefly, the intention-to-treat (ITT) set comprises all registered participants except for prespecified exclusions (e.g., ineligibility identified after registration or major GCP non-compliance) and requires an available reference-standard diagnosis. The full analysis set (FAS) further excludes cases with missing standard work-up components or non-evaluable DCR images. The alternative FAS (alt-FAS) for six-zone agreement includes cases with evaluable DCR and perfusion scintigraphy images and an interval <15 days between the two examinations. Cases lacking a reference-standard diagnosis will not contribute to the primary analysis but will be accounted for in the participant flow diagram with reasons. Non-evaluable reads due to non-diagnostic image quality will be summarized; for the primary accuracy endpoint, indeterminate reads will be treated as incorrect in a prespecified sensitivity analysis. No imputation is planned.

### Monitoring, auditing, quality control, and safety

Study monitoring is planned and will be conducted in accordance with a prespecified monitoring plan to ensure that the trial is performed safely and in compliance with the protocol, applicable regulations, and Good Clinical Practice for medical device trials. Monitoring will include on-site and/or off-site procedures, such as source data verification and review of essential trial documents, as appropriate.

Audits are planned to be conducted at least once annually. At the time of manuscript submission, a detailed audit procedure has not yet been finalized; however, audits will be performed by personnel independent of the study conduct and monitoring activities, and will assess compliance with the protocol and applicable regulatory requirements. Adverse events during DCR and device malfunctions will be recorded and reported according to sponsor procedures and regulatory requirements. Periodic safety and progress reports will be submitted to the institutional review board (IRB).

## Discussion

This protocol addresses a structural limitation of current CTEPH diagnostic pathways: reliance on V/Q scintigraphy, which may be delayed or unavailable outside tertiary centers. By embedding DCR-an X-ray-based technique potentially accessible in a wide range of hospitals-into the initial assessment, the study tests whether regional perfusion surrogates can improve early discrimination of CTEPH and prioritize referral for definitive testing.

The multi-reader, case-wise randomized controlled design aims to isolate the diagnostic contribution of DCR while minimizing incorporation bias. By adhering to STARD recommendations, using κ-based six-zone regional agreement, and adopting multi-reader multi-case inference, the study seeks to generate robust and transportable performance estimates. Although the primary endpoint was defined as a confirmatory outcome in agreement with the PMDA, we also designed the analysis and reporting to comply with STARD 2015 as a key academic framework, thereby maximizing the scientific transparency and interpretability of this diagnostic accuracy study.

Important limitations include the need for reader training to standardize DCR interpretation and the technical skills required for DCR image acquisition. Even if DCR does not demonstrate superiority, the study will delineate realistic performance boundaries for DCR and help define future hybrid diagnostic pathways, such as DCR-first strategies followed by V/Q scintigraphy in equivocal cases [14]. Another limitation is that patients with symptomatic acute pulmonary embolism within 3 months before enrollment are excluded to allow clearer evaluation of diagnostic performance in a single target disease entity. In addition, because this study enrolls patients with echocardiographically suspected pulmonary hypertension, the results may not be fully generalizable to acute-on-chronic presentations or to patients with less advanced chronic thromboembolic disease, including those with relatively preserved right ventricular structure/function or a lower echocardiographic probability of pulmonary hypertension.

### Patient and public involvement

Representatives of patients living with pulmonary arterial hypertension were involved in transparency and information-sharing activities for this trial. Specifically, we shared non-technical information on the study design and conduct with the Japanese non-profit patient organization “PHA Japan” to enhance openness and understanding of the trial among patients and their families. Patients and the public were not directly involved in the choice of endpoints, eligibility criteria, or recruitment procedures, but their perspectives informed how the study is communicated to potential participants and the wider community.

### Dissemination

Results will be submitted to peer-reviewed journals and presented at scientific meetings.

### Trial status

This manuscript is based on version 3.2 of the clinical investigation plan dated 29/09/2025. The first patient was enrolled on 31/07/2025. Recruitment started on 30/07/2025 and is ongoing, with enrollment expected to be completed by 28/02/2027 and overall study completion planned for 31/05/2027. We anticipate that primary results will be available after study completion (expected in mid-2027).

## Supporting information

S1 Checklist SPIRIT checklist. Completed SPIRIT checklist for the FIND-DCR study.
(DOCX)

S1 Protocol Original study protocol. Original English version of the study protocol approved by the ethics committee.
(PDF)

S1 File Sample DCR-based pulmonary circulation movie. Sample movie of DCR-based pulmonary circulation analysis.
(MP4)
