# Supplementary material for: Facilitating early diagnosis of chronic thromboembolic pulmonary hypertension with dynamic chest radiography: Protocol for a multicenter, assessor-blinded, case-wise randomized superiority reader study (FIND-DCR)
Source: PLoS One. 2026 Jun 11;21(6):e0350858. doi: 10.1371/journal.pone.0350858 (PMC13258118; doi:10.1371/journal.pone.0350858)
Supplement: S1 Checklist — Completed SPIRIT checklist for the FIND-DCR study. (DOCX) [file pone.0350858.s001.docx]

This study is a prospective diagnostic accuracy study with a case-wise randomized reader design. Items not applicable to therapeutic randomized controlled trials are marked as “Not applicable,” and the study is designed and reported in accordance with the STARD 2015 guideline.

| **Item** | **Description** | **Applicable?** | **Where addressed** |
| --- | --- | --- | --- |
| 1 | Title | Yes | Title page |
| 2a | Trial registration | Yes | Trial registration section |
| 2b | WHO Trial Registration Data Set | Yes | Trial registration section |
| 3 | Protocol version | Yes | Protocol version and date |
| 4 | Funding | Yes | Funding section |
| 5 | Roles and responsibilities | Yes | Author contributions |
| 6a | Rationale and background | Yes | Introduction |
| 6b | Explanation for choice of comparators | Yes | Methods – Study design |
| 7 | Objectives | Yes | Objectives and hypotheses |
| 8 | Trial design | Yes | Methods – Study design |
| 9 | Study setting | Yes | Methods – Study design and setting |
| 10 | Eligibility criteria | Yes | Methods – Participants |
| 11a | Interventions | **Not applicable** | Diagnostic accuracy study (no therapeutic intervention) |
| 11b | Criteria for discontinuing interventions | **Not applicable** | Diagnostic imaging study |
| 12 | Outcomes | Yes | Outcomes section |
| 13 | Participant timeline | Yes | **Table 3** |
| 14 | Sample size | Yes | Sample size |
| 15 | Recruitment | Yes | Trial status |
| 16 | Allocation | Yes | Randomisation |
| 17 | Blinding | Yes | Blinding |
| 18 | Data collection methods | Yes | Data collection and management |
| 19 | Data management | Yes | Data management |
| 20 | Statistical methods | Yes | Statistical analysis |
| 21 | Monitoring | Yes | Methods: Monitoring and auditing |
| 22 | Harms | Yes | Safety endpoints |
| 23 | Auditing | Yes | Methods: Monitoring and auditing |
| 24 | Research ethics approval | Yes | Ethics and dissemination |
| 25 | Protocol amendments | Yes | Ethics section |
| 26 | Consent | Yes | Ethics section |
| 27 | Confidentiality | Yes | Ethics section |
| 28 | Declaration of interests | Yes | Competing interests |
| 29 | Access to data | Yes | Data availability |
| 30 | Dissemination policy | Yes | Ethics and dissemination |
