# Supplementary material for: Facilitating early diagnosis of chronic thromboembolic pulmonary hypertension with dynamic chest radiography: Protocol for a multicenter, assessor-blinded, case-wise randomized superiority reader study (FIND-DCR)
Source: PLoS One. 2026 Jun 11;21(6):e0350858. doi: 10.1371/journal.pone.0350858 (PMC13258118; doi:10.1371/journal.pone.0350858)
Supplement: S1 Protocol — Original English version of the study protocol approved by the ethics committee. (PDF) [file pone.0350858.s002.pdf]

Facilitating Diagnosis of CTEPH With Dynamic Chest Radiography: An Investigator-led, Multicenter, Assessor-blinded, Case-wise Randomized, Superiority Reader Study in Patients With Echocardiographically Suspected Pulmonary Hypertension, Comparing Standard Initial Workup (Blood Test, Chest X-ray, ECG, Pulmonary Function Test) Plus A DCR-based Pulmonary Circulation Analysis Program with Standard Initial Workup alone for Early Diagnosis of Chronic Thromboembolic Pulmonary Hypertension — The FIND-DCR Trial

Protocol No.: CTR340-01

Coordinating Investigator: Kota Abe, MD, PhD

Department of Cardiovascular Medicine, Faculty of Medical Sciences, Kyushu University

Version: 3.2

Date: 29 September 2025

1 Introduction

1.1 Chronic thromboembolic pulmonary hypertension

Chronic thromboembolic pulmonary disease (CTEPD) is a condition in which chronic obstruction of the pulmonary arteries by organised thrombi results in a fixed abnormal distribution of pulmonary blood flow and abnormal pulmonary haemodynamics that persist for more than three months. CTEPH is defined as CTEPD complicated by pulmonary hypertension, with mean pulmonary artery pressure (mPAP) ≥25 mmHg (definition updated to >20 mmHg in the 2022 ESC/ERS guideline; Japanese guidelines are expected to be updated accordingly in 2025).[1–3] CTEPH is a designated intractable disease in Japan. In fiscal year 2019, the number of recipients of the Specific Medical Expense Subsidy (Designated Intractable Diseases) certificate for CTEPH was 4,169, an increase of 370 from the previous year. The male-to-female ratio is approximately 1:1.7, and the mean age is 64 years.[4] CTEPH is characterised by pulmonary artery obstruction due to organised intraluminal thrombi and the resultant pulmonary hypertension and is classified as Group 4 pulmonary hypertension. Major symptoms include dyspnoea, easy fatigability, palpitations, chest pain, back pain, cough, haemoptysis and syncope. With conservative therapy alone, pulmonary arterial pressure increases and can progress to right heart failure; the 5-year survival rate is approximately 40%, indicating a poor prognosis.[1–3]

The pathogenesis of CTEPH is thought to involve progression from recurrent or occult acute pulmonary thromboembolism; however, the underlying mechanisms are not fully understood. Thrombi derived from patients with CTEPH have been reported to be less susceptible to lysis than thrombi from healthy individuals. Recurrent embolisation and thrombus propagation within the pulmonary arteries may lead to chronic obstruction and stenosis, and secondary microvascular disease may further contribute to the progression of pulmonary hypertension.[1–3]

1.2 Current diagnostic approach for CTEPH

According to the 2015 European Society of Cardiology/European Respiratory Society (ESC/ERS) Guidelines for the diagnosis and treatment of pulmonary hypertension,[1] and the major Japanese guidelines for CTEPH practice (Guidelines for the Treatment of Pulmonary Hypertension by the Japanese Circulation Society, 2017; and the CTEPH Clinical Practice Guidelines by the Japanese Pulmonary Circulation and Pulmonary Hypertension Society, 2022),[2,3] when pulmonary hypertension is suspected based on medical history, physical findings and other information, differential diagnosis should be pursued using transthoracic echocardiography in addition to the initial work-up (blood tests, electrocardiography, chest radiography and pulmonary function tests). When pulmonary hypertension is strongly suspected on transthoracic echocardiography and the initial work-up suggests that common lung disease and left heart disease are the primary cause and the likelihood of CTEPH (or concomitant CTEPH) is low, specialised tests such as high-resolution CT (HRCT) or coronary CT angiography may be performed. If suspicion for CTEPH remains, CT pulmonary angiography and/or ventilation–perfusion (V/Q) scintigraphy are performed.[1–3] In particular, V/Q scintigraphy is currently the only test that evaluates pulmonary circulation, and it has high sensitivity and specificity (90–100% and 94–100%, respectively).[1–3,7,8] Therefore, for

early diagnosis it is important to access V/Q scintigraphy promptly once pulmonary hypertension is suspected on transthoracic echocardiography.[1–3] For patients in whom a perfusion defect is identified on perfusion scintigraphy, prompt referral to a CTEPH expert centre is recommended so that specialised interventions can be provided without delay, including medical treatment (pulmonary vasodilator therapy), pulmonary endarterectomy and balloon pulmonary angioplasty (BPA).[1–3]

1.3 Rationale for this clinical trial

CTEPH with mPAP >30 mmHg generally has an extremely poor prognosis due to progression of right heart failure, acute exacerbation of respiratory failure such as pneumonia, and recurrence of acute pulmonary embolism even when minor; early therapeutic intervention is essential (Figure 1).[5]

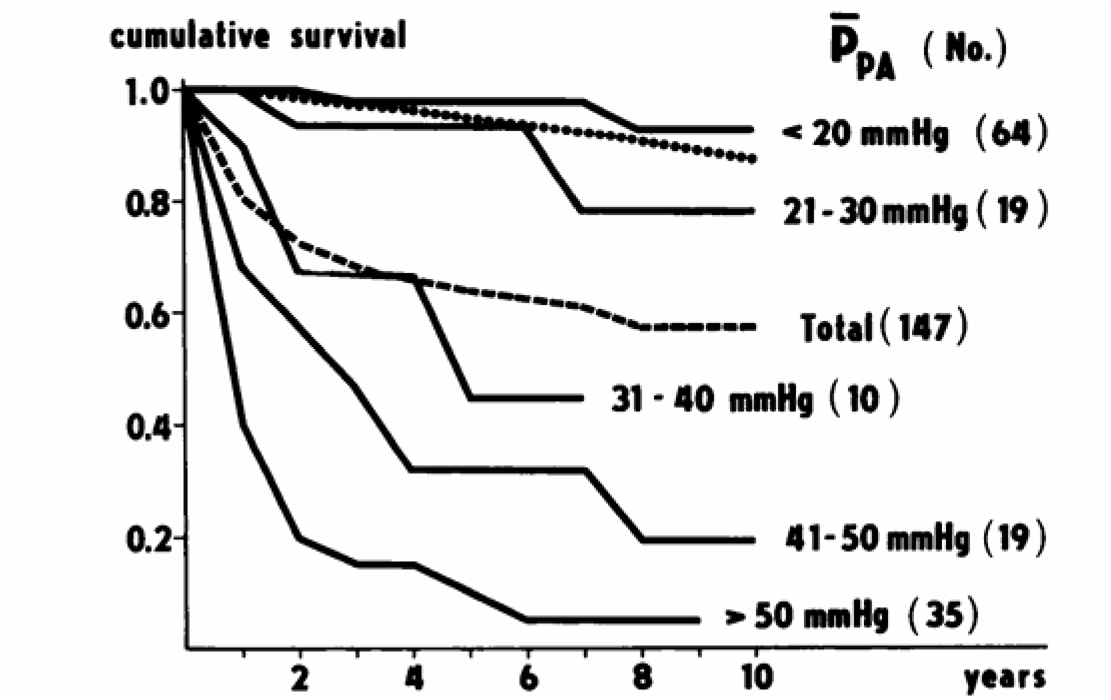

Figure 1. Prognosis of CTEPH according to pulmonary arterial pressure

Conversely, it has been reported that substantial time is required to reach a definitive diagnosis of CTEPH. In a retrospective study of 33 patients with CTEPH conducted at Kyoto University Hospital and Shinshu University Hospital, the time from symptom onset to definitive diagnosis of CTEPH was approximately 32 months (median; interquartile range 14–81 months); the time from first presentation to a healthcare facility to definitive diagnosis was 20 months (median; interquartile range 6–57 months); and the time from first suspicion of pulmonary hypertension to definitive diagnosis was 7 months (median; interquartile range 2–28 months) (Figure 2).[6] These data suggest that while some patients receive a definitive diagnosis within approximately 2 months after pulmonary hypertension is suspected, others may require more than 2 years.

One reason for diagnostic delay is the low availability of nuclear medicine equipment in general clinics and non-expert centres where patients are first assessed. Nuclear medicine scanners required for V/Q scintigraphy are installed only in limited facilities. In 2020, the installation rate of nuclear medicine equipment by hospital size was 95% in advanced treatment hospitals such as university hospitals (which comprise the majority of CTEPH expert centres) but was 40% in regional support hospitals with ≥200 beds, 4% in general hospitals with <200 beds, and 0% in general clinics.[9,10]

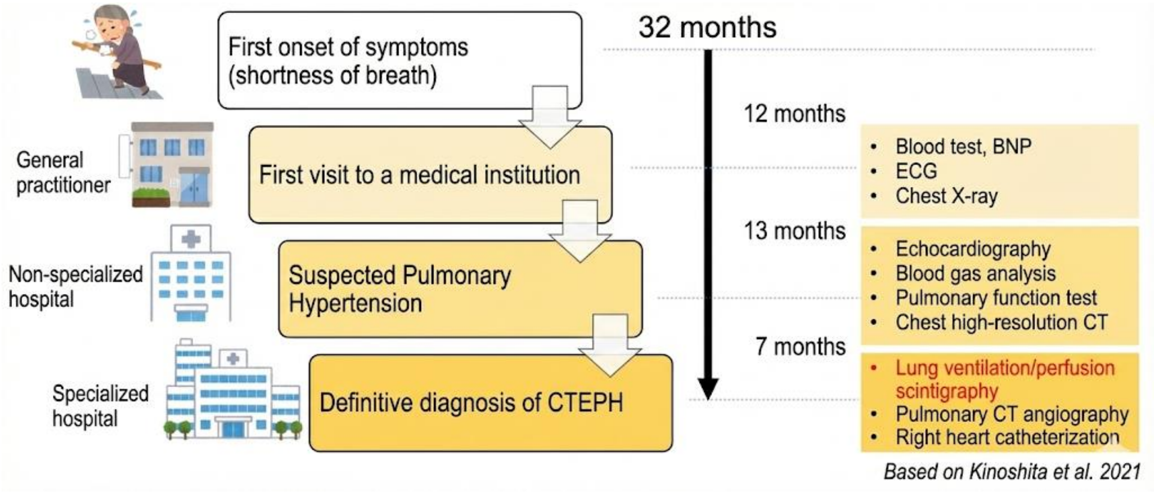

Figure 2. Time lapse to definitive diagnosis of CTEPH

Although guidelines require V/Q scintigraphy to evaluate pulmonary perfusion impairment, many non-expert centres (e.g., regional support hospitals) cannot perform this examination and must refer patients to advanced hospitals. When patients cannot promptly visit a CTEPH expert centre due to distance or personal circumstances, some undergo only contrast-enhanced CT to confirm the absence of central thrombi; this may miss lesions distal to segmental branches (peripheral-type CTEPH). In addition, indiscriminate use of pulmonary vasodilators may further delay referral. In other words, there is a substantial accessibility gap between transthoracic echocardiography/chest radiography and V/Q scintigraphy, which constitutes a major barrier to CTEPH diagnosis. The investigational device is expected to serve as an innovative, simple and non-invasive tool that fills this accessibility gap. By enabling easy assessment of pulmonary perfusion impairment even in non-expert centres, it may support clinicians in judging the urgency and priority of referral to expert centres and may contribute to earlier treatment intervention and, ultimately, improved prognosis.

In a questionnaire survey on V/Q scintigraphy conducted in November 2022 among physicians specialising in CTEPH at 35 advanced treatment hospitals affiliated with the Japanese Pulmonary Circulation and Pulmonary Hypertension Society, the waiting period for V/Q scintigraphy in their own expert centres was found to be 7 days to 1 month because radiopharmaceuticals must be procured for each use. Among the 35 expert centres that responded (34/35), many indicated that a diagnostic device capable of evaluating pulmonary perfusion on the same day is needed, as referred patients from distant areas would otherwise need to return solely for the examination. Thus, accessibility gaps to V/Q

scintigraphy also exist even in expert centres, and the investigational device may be useful as an immediate and convenient method for evaluating pulmonary perfusion even in such centres (Figure 3).

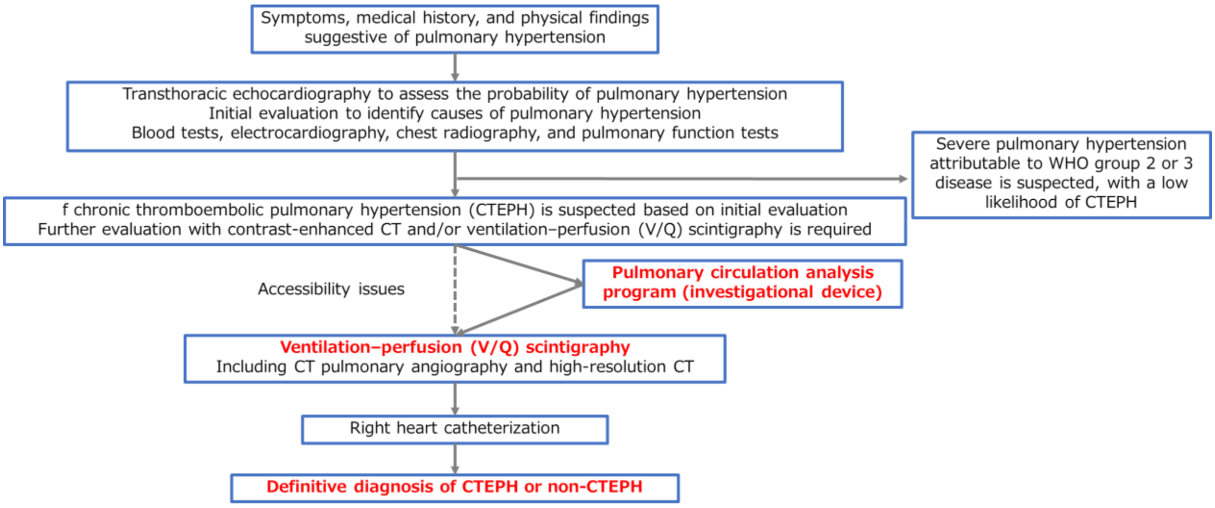

Figure 3. Clinical position of this test device in terms of clinical practice guidelines (Partially revised from the 2017 revision of the Pulmonary Hypertension Treatment Guidelines to reflect actual medical treatment)

Because V/Q scintigraphy requires more than 1.5 hours of examination time when preparation and radiopharmaceutical administration are included, it imposes a substantial burden on patients. For this reason, alternative approaches using chest magnetic resonance imaging (MRI) have been developed and are under investigation overseas.[11,12]

Dynamic X-ray imaging is a sequence of X-ray images acquired by irradiating pulsed X-rays (15 frames per second) for a fixed duration (minimum 6 seconds to maximum 10 seconds). In recent years, flat panel detectors (FPDs) with high X-ray detection efficiency have been developed, enabling dynamic X-ray imaging at radiation doses comparable to conventional static radiography.[13] (See Section 4, Overview of the investigational device.) The pulmonary circulation analysis program used in this trial analyses dynamic chest radiography acquired by the dynamic imaging system. By detecting temporal changes in X-ray transmittance corresponding to pulsatile changes in pulmonary blood volume, the program outputs information related to pulmonary blood flow and displays it on an external device.

The principle for measuring local pulmonary blood flow information has been demonstrated in animal models of pulmonary embolism, showing that reduced blood flow due to pulmonary artery embolisation can be detected as a decrease in signal change on dynamic X-ray images.[14,15] Clinically, case reports in patients with pulmonary thrombosis have also been published.[16,17] In a retrospective study of 50 patients with pulmonary hypertension (29 with CTEPH and 21 with non-CTEPH) conducted at Kyushu University Hospital, high diagnostic agreement for CTEPH was demonstrated between V/Q scintigraphy and dynamic chest radiography/pulmonary circulation analysis (see Section 1.4 for sample size rationale).[18] Thus, by analysing changes in pixel values, evaluation of pulmonary perfusion impairment

similar to perfusion scintigraphy is feasible. By adding this pulmonary circulation analysis program to chest X-ray systems capable of dynamic imaging, pulmonary perfusion impairment can be assessed and simple discrimination between CTEPH and non-CTEPH can be performed even in facilities without scintigraphy. This may facilitate rapid referral to expert centres and enable earlier follow-up examinations such as V/Q scintigraphy, thereby contributing to earlier diagnosis and earlier intervention for CTEPH.

Although V/Q scintigraphy has extremely high diagnostic performance (sensitivity and specificity 90–100% and 94–100%, respectively) and the investigational device is not intended to fully replace it, the convenience and immediacy of the pulmonary circulation analysis program may increase the pre-test probability of CTEPH when added to the initial work-up, with substantial clinical significance. Accordingly, this trial was planned to test whether adding dynamic chest radiography/pulmonary circulation analysis program to the initial work-up improves diagnostic accuracy for discriminating CTEPH from non-CTEPH, and to demonstrate superiority of initial diagnostic accuracy.

Note 1: A flat panel detector (FPD) is a device that receives X-rays transmitted through the body and converts them into electrical signals to obtain digital X-ray images.

1.4 Comparison of V/Q scintigraphy, dynamic chest radiography/pulmonary circulation analysis program and static chest radiography

In V/Q scintigraphy, when the ventilation scan shows no abnormal findings (areas of decreased uptake) and the perfusion scan shows a segmental defect or multiple subsegmental defects (areas of decreased uptake), pulmonary perfusion impairment is diagnosed.

In dynamic chest radiography/pulmonary circulation analysis, when no parenchymal abnormality is identified on the static chest radiograph but a wedge-shaped segmental defect suggestive of a regional pattern is present on the pulmonary circulation analysis images, pulmonary perfusion impairment is diagnosed.

A comparison of V/Q scintigraphy, dynamic chest radiography/pulmonary circulation analysis and static chest radiography (e.g. conventional chest X-ray) is shown in Table 1.

Table 1. The diagnostic performance of V/Q scintigraphy, DCR-based pulmonary circulation analysis, and chest X-ray

	V/Q scintigraphy	Dynamic chest radiography / pulmonary circulation analysis program	Chest X-ray
Evaluation focus	Pulmonary arterial perfusion impairment	Pulmonary vascular perfusion impairment	Assessment of interruption of pulmonary vascular shadows/diameter

			and presence of pulmonary infarction shadow
Diagnostic performance for CTEPH	Extremely high*	High**	Low***
Spatial resolution	Central to subsegmental to peripheral	Central to subsegmental	Central to segmental branches
Immediacy	No	Yes	Yes
Invasiveness	Intravenous injection of radiopharmaceuticals	None	None
Radiation exposure	1.2–2.0 mSv	0.1–0.4 mSv	0.1 mSv
Cost	High	Low	Low
Repeatability	Limited	Yes	Yes
Key limitations	Long examination time; radiopharmaceuticals and large-scale equipment required	No evidence from prospective randomised controlled trials	Low diagnostic performance

* The diagnostic performance of V/Q scintigraphy is extremely high, with sensitivity and specificity of 90–100% and 94–100%, respectively.[1,3,7,8]

** The diagnostic performance of dynamic chest radiography/pulmonary circulation analysis was high in a retrospective study (see Section 1.5 and Table 2).[18]

*** The diagnostic performance of static chest radiography is low (see Table 2).[19]

1.5 Study evaluating the performance of the pulmonary circulation analysis program as an instrument for assessing perfusion impairment

To evaluate the performance of the pulmonary circulation analysis program, a retrospective study was conducted at Kyushu University Hospital in 50 patients with pulmonary hypertension who underwent V/Q scintigraphy and dynamic chest radiography (29 with CTEPH and 21 with non-CTEPH). The interval between V/Q scintigraphy and dynamic chest radiography was within 2 months. V/Q scintigraphy was interpreted by consensus of two nuclear medicine specialists, and dynamic chest radiography/pulmonary circulation analysis images were interpreted by consensus of two board-certified radiologists.[18]

The agreement between V/Q scintigraphy and the pulmonary circulation analysis program for evaluation of pulmonary perfusion impairment in CTEPH was 0.90 [0.79–0.95], and the kappa coefficient was 0.79 [0.61–0.96], indicating strong agreement. In addition, the presence of regional perfusion defects was evaluated by dividing each lung into upper, middle and lower regions (six regions in total) (Table 2). CTEPH was defined as the presence of at least one regional perfusion defect on each modality. Sensitivity, specificity, positive predictive value (PPV), negative predictive value (NPV), accuracy and the area under the curve (AUC) were calculated. For comparisons, the McNemar test was used for sensitivity,

specificity and accuracy, and the DeLong method was used for AUC. There were no significant differences between V/Q scintigraphy and dynamic chest radiography/pulmonary circulation analysis in sensitivity, specificity, accuracy or AUC.

Table 2. Comparison of diagnostic performance in the retrospective study.18,19

	Sensitivity (%, 95% CI)	Specificity (%, 95% CI)	PPV (%, 95% CI)	NPV (%, 95% CI)	Accuracy (%, 95% CI)	AUC
Static chest radiography, Conventional chest X-ray*	39.2 (73/186) [33.9–44.4]	78.5 (212/270) [74.9–82.1]	55.7 (73/131) [48.2–63.0]	65.2 (212/325) [62.2–68.2]	62.5 (285/456) [58.2–66.7]	0.65
Dynamic chest radiography / pulmonary circulation analysis program	96.6 (28/29) [87.5–99.3]	85.7 (18/21) [73.2–89.6]	90.3 (28/31) [81.9–92.9]	94.7 (18/19) [80.9–99.0]	92.0 (46/50) [81.5–95.2]	0.92
V/Q scintigraphy	100 (29/29) [92.1–100]	85.7 (18/21) [74.7–85.7]	90.6 (29/32) [83.4–90.6]	100 (18/18) [87.2–100]	94.0 (47/50) [84.8–94.0]	0.93
P value	0.32	1.00	-	-	0.65	0.85

* Static chest radiography performance is based on the non-public dataset described in Reference 19.

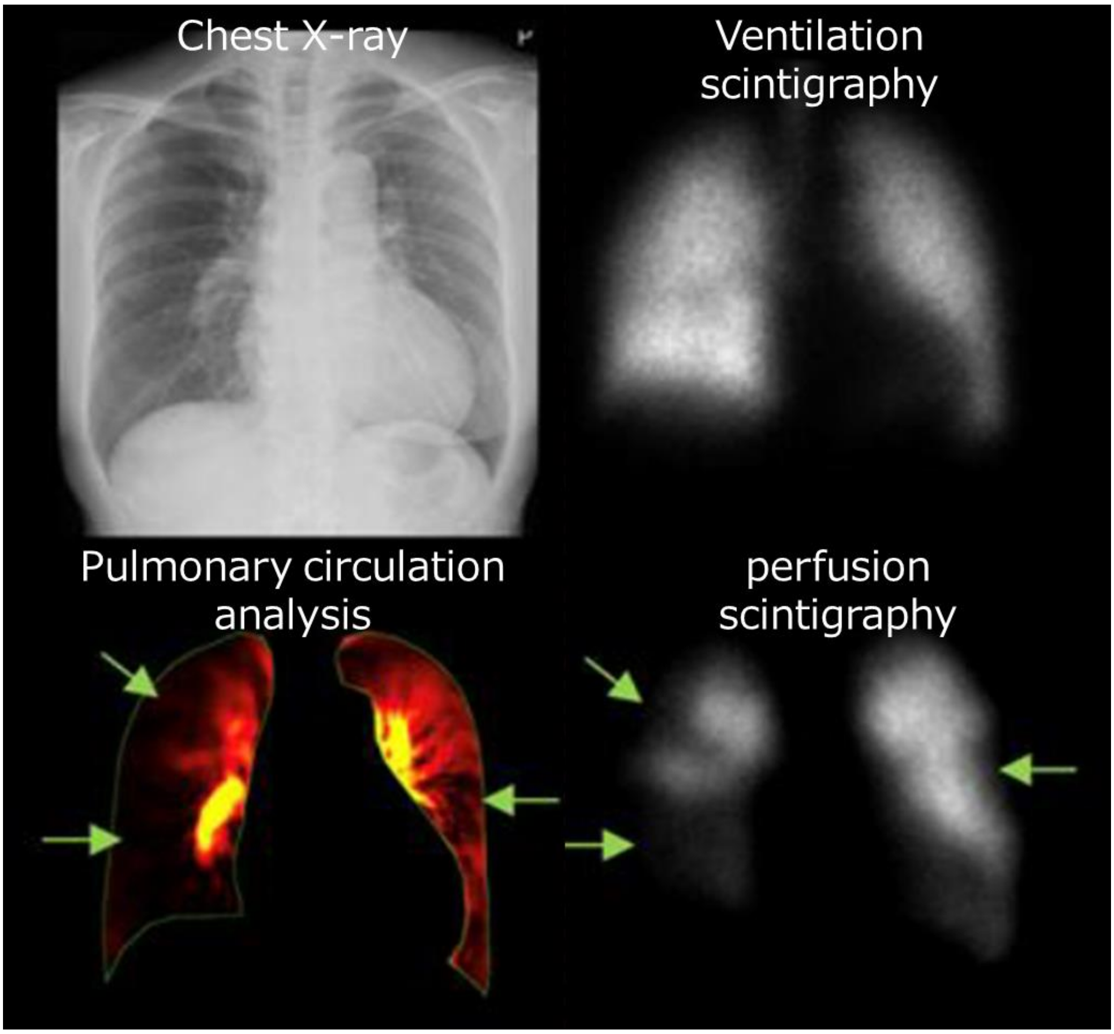

Figure 4: Typical imaging findings of a patient with CTEPH. No abnormality is evident on static chest radiography and ventilation scintigraphy, whereas a similar segmental pulmonary perfusion defect is observed on dynamic chest radiography/pulmonary circulation analysis images and on perfusion scintigraphy.

2 Objective

This trial aims to verify the superiority of diagnostic accuracy for discriminating CTEPH from non-CTEPH (diseases other than CTEPH) when dynamic chest radiography/pulmonary circulation analysis is added to the standard initial work-up, compared with the standard initial work-up alone.

3 Eligibility criteria

3.1 Inclusion criteria

- Male or female aged ≥18 years at the time of informed consent.

- Patients who, in accordance with the pulmonary hypertension diagnostic algorithm (Japanese Circulation Society: Guidelines for the Treatment of Pulmonary Hypertension

[2017 revision]),[2] are considered to have a high probability of pulmonary hypertension on transthoracic echocardiography (High probability in the echocardiographic assessment of PH; see Tables 3 and 4), and for whom V/Q scintigraphy and right heart catheterisation are scheduled for discrimination and definitive diagnosis of CTEPH versus non-CTEPH.

- Patients who have received sufficient explanation from the treating physician about this trial, have understood the information, and have provided written informed consent voluntarily.

Note: Transthoracic echocardiography

In the 2017 Guidelines for the Treatment of Pulmonary Hypertension (Japanese Circulation Society),2 it is recommended that peak tricuspid regurgitation velocity itself be used for screening (Table 3). A peak tricuspid regurgitation velocity >3.4 m/s strongly suggests pulmonary hypertension and meets the eligibility criterion for this trial. In addition, if peak tricuspid regurgitation velocity is ≥2.9 m/s and at least one echocardiographic finding suggestive of pulmonary hypertension is present—such as right ventricular enlargement, interventricular septal flattening due to an enlarged right ventricle, right atrial enlargement, or pulmonary artery flow velocity waveform changes—pulmonary hypertension should be strongly suspected and the patient meets the eligibility criterion for this trial (Table 4).

Table 3. Transthoracic echocardiography in patients with suspected pulmonary hypertension[2]

Tricuspid regurgitation peak velocity (TRV)	Other echocardiographic signs suggestive of PH	Echocardiographic probability of PH
≤ 2.8 m/s, or not measurable	Absent	Low
≤ 2.8 m/s, or not measurable	Present	Intermediate
2.9–3.4 m/s	Absent	Intermediate
2.9–3.4 m/s	Present	High (eligible for this trial)
> 3.4 m/s	Not required	High

Abbreviations: PH, pulmonary hypertension; TRV, tricuspid regurgitation peak velocity. **Note:** “Not measurable” indicates that TRV could not be measured on echocardiography.

Table 4. Echocardiographic signs suggestive of pulmonary hypertension (PH)[2]

Site assessed	Findings

Site assessed	Findings
Right ventricle and left ventricle	RV/LV basal diameter ratio > 1.0
	Interventricular septal flattening (particularly LV eccentricity index > 1.1 in systole)
Right atrium	End-systolic right atrial area (apical four-chamber view) > 18 cm^2
Inferior vena cava	IVC diameter > 21 mm with reduced inspiratory collapse (<50% with a sniff and <20% with quiet inspiration)
Pericardial effusion	Presence of pericardial effusion
Pulmonary artery	RV outflow tract systolic acceleration time < 105 ms, or a biphasic waveform (mid-systolic notching)
	Early diastolic pulmonary regurgitation velocity > 2.2 m/s
	Pulmonary artery diameter > 25 mm

Abbreviations: PH, pulmonary hypertension; RV, right ventricle; LV, left ventricle; IVC, inferior vena cava; RVOT, right ventricular outflow tract.

3.2 Exclusion criteria

- At screening, severe obstructive lung disease with FEV1.0 <60% of predicted, or severe restrictive lung disease with total lung capacity (TLC) <60% of predicted; or pulmonary hypertension due to lung disease (Group 3 pulmonary hypertension) judged by the principal or subinvestigator to adequately explain the pulmonary hypertension.

- At screening, severe valvular disease of the aortic valve or mitral valve, or left ventricular ejection fraction (LVEF) <40% on transthoracic echocardiography; or pulmonary hypertension due to left heart disease (Group 2 pulmonary hypertension) judged by the principal or subinvestigator to adequately explain the pulmonary hypertension.

- Poorly controlled tachyarrhythmia (resting heart rate >100 bpm).

- Diagnosis of acute symptomatic pulmonary embolism on imaging within 3 months prior to registration.

- Congenital or acquired abnormalities of pulmonary vascular anatomy or course.

- History of lung resection or radiotherapy to the chest.

- Active thoracic malignancy.

- History of vasculitis.

- Presence of devices in the chest (e.g., pacemakers or implantable cardioverter-defibrillators) for which pulsed X-ray imaging may be contraindicated and/or that may interfere with dynamic chest radiography and image analysis.

- Women who are pregnant, or women of childbearing potential who are not using appropriate contraception.

- Any other condition judged by the principal or subinvestigator to make the patient unsuitable for participation in this trial.

3.3 Discontinuation criteria

- Discontinuation judged necessary by the principal investigator (e.g., serious adverse events related to the investigational device, poor subject compliance).

- Request by the subject to discontinue participation.

4 Overview of the investigational device

4.1 Regulatory classification of the investigational device

- Category: diagnostic program (disease diagnosis program).

- Generic name: program for workstation for X-ray diagnostic imaging devices.

- Device class: Class II.

- Name of investigational device: Pulmonary Circulation Analysis Program.

- In this trial, we will use "PH2-MODE" installed on the "Diagnostic Imaging Workstation KONICA MINOLTA DI-X1" (Marketing authorisation certification No.: 230ABBZX00092000).

- Identification code: CTR340.

4.2 X-ray static/dynamic imaging system

- A stationary digital general-purpose X-ray diagnostic apparatus, or a mobile digital general-purpose X-ray diagnostic apparatus.

- Product name: Diagnostic X-ray system RADspeed Pro (SHIMADZU Corp. Tokyo, Japan).

- Marketing authorisation certification No.: 221ABBZX00210000.

- Digital radiograph with flat panel detector output reading.

- Product name: Digital Radiography SKR 3000 (KONICA MINOLTA v Tokyo, Japan).

- Marketing authorisation certification No.: 228ABBZX00115000.

- Within the basic configuration: "DR panel".

Clinical trial devices and other equipment used in the trial

In this trial, the clinical trial devices consist of the investigational device and approved imaging devices used to acquire and analyse dynamic chest radiography images. In addition, standard-of-care systems for lung ventilation–perfusion (V/Q) scintigraphy and image viewing are used.

Clinical trial devices

Investigational device

- Name: Pulmonary Circulation Analysis Program (PH2-MODE) installed on the image diagnostic workstation “KONICA MINOLTA DI-X1” (Marketing authorisation certification No.: 230ABBZX00092000).

- Identification code: CTR340.

Other clinical trial devices (approved medical devices already installed at participating sites)

- X-ray static/dynamic imaging system (e.g., RADspeed Pro; SKR 3000; DR panel; image-processing controller).

- These devices will be managed and maintained in accordance with each participating site’s procedures and applicable regulations.

Other devices used in the trial

- Lung V/Q scintigraphy imaging system: a rotating gamma camera SPECT system or SPECT/CT system used for routine clinical examinations at each participating site.

- Image diagnosis system (viewer): a nuclear medicine image-processing workstation and/or a general-purpose diagnostic image viewer. Images should be reviewed on a monitor that meets JIRA “JESRA X-0093*B-2017” management grade 1A (maximum luminance ≥350 cd/m²).

System configuration and data flow

Dynamic chest radiography images are acquired by irradiating pulsed X-rays (15 frames per second) for a fixed duration (minimum 6 s to maximum 10 s) to generate a sequence of chest radiographs. The acquired dynamic images are submitted to the image interpretation committee via the upload system, analysed on the DI-X1 workstation using PH2-MODE (KONICA MINOLTA INC. Tokyo, Japan), and the resulting pulmonary circulation analysis images are transmitted to the diagnostic viewer for interpretation.

For V/Q scintigraphy, planar images, SPECT images, and (when available) CT images for fusion are acquired using the SPECT or SPECT/CT system. The images are submitted to the image interpretation committee via the upload system, processed on a nuclear medicine image-processing workstation, and transmitted to the diagnostic viewer for interpretation.

Method for extracting pulmonary blood-flow information using the Pulmonary Circulation Analysis Program

As shown in Figures 6, pulmonary arterial blood volume changes with cardiac pulsation, which leads to temporal changes in pixel values in dynamic chest radiography images. The investigational device detects and visualises pulmonary blood-volume change from end-diastole by the following steps:

- Detect the time course of pixel values within a region of interest (ROI) placed over the heart, PV_H(frame), and estimate the cardiac frequency f0 from PV_H(t).

- Detect the time course of pixel values within multiple ROIs placed in the lung fields, PV_L(x, y, frame).

- Apply a band-pass filter that passes only the frequency band around f0 to PV_H(frame) and PV_L(x, y, frame) to remove signal components not related to cardiac pulsation; the filtered signals are denoted FPV_H(frame) and FPV_L(x, y, frame).

- Detect the end-diastolic frame Fd from FPV_H(frame) (reference frame). For each frame, compute the change in the lung-field signal relative to the reference frame:

ΔFPV_L(x, y, frame) = FPV_L(x, y, frame) − FPV_L(x, y, Fd)

- Colour-code ΔFPV_L(x, y, frame) for each frame using a predefined colour lookup table to generate a cine sequence (pulmonary circulation analysis movie: PH2-Dynamic).

- Generate a minimum intensity projection (MinIP) image from the cine sequence as a representative image (pulmonary circulation analysis summary image: PH2-Summary).

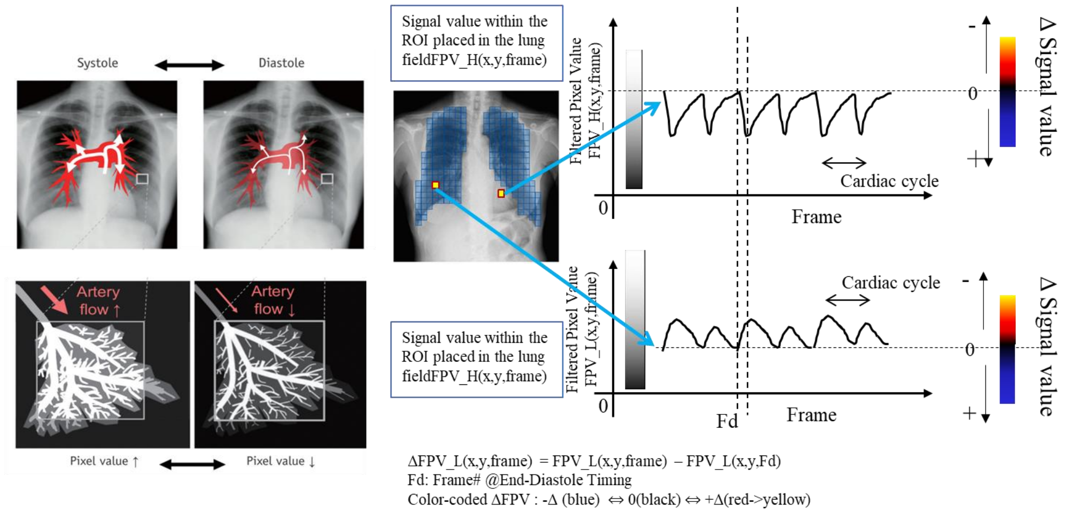

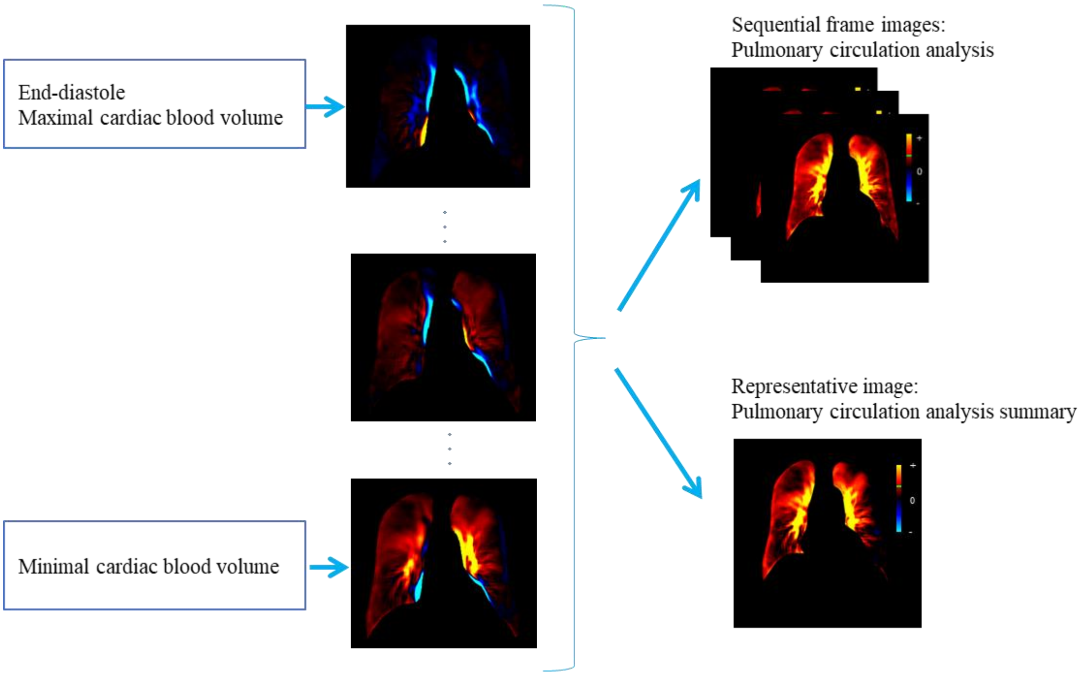

Figure 6. Operating Principles of the Pulmonary Circulation Analysis Program[13-14]

Input, output, and interpretation of the investigational device

Input

Expected acquisition conditions for dynamic chest radiography

- Patient position and projection: supine anteroposterior (AP; recommended) or upright posteroanterior (PA).

- Breath-hold: 6–10 s (typically 7 s).

- Pulsed X-ray parameters: tube voltage and mAs are set according to the participant's BMI so that the signal-to-noise ratio of the dynamic images is maintained above a prespecified threshold as far as possible irrespective of body mass index.

- Frame rate: 15 frames per second.

- Image size: 17 × 17 in (424.8 × 424.8 mm; 1,062 × 1,062 pixels; 400 µm pitch) or 14 × 17 in (348.8 × 425.6 mm; 872 × 1,064 pixels; 400 µm pitch).

Rationale: In Protocol Version 3.0, upright PA acquisition was permitted. A previous report suggested comparable diagnostic performance between supine and upright acquisition for acute pulmonary embolism (Yamasaki et al., Eur J Radiol Open 2024;100602).

Output

- ΔFPV_L(x, y, frame): frame-by-frame change in the filtered lung-field signal relative to the reference frame.

- Note: a filter that passes only the frequency band around the cardiac cycle is applied in advance to extract pulsation-related signal changes.

- PH2-Dynamic: a colour-coded cine sequence generated by applying a predefined colour table to ΔFPV_L(x, y, frame).

- PH2-Summary: a representative image generated from PH2-Dynamic using minimum intensity projection (MinIP).

Interpretation and handling of the output

- Regions with a small ΔFPV_L(x, y, frame) appear darker on PH2-Dynamic/PH2-Summary.

- A small pulsation-related signal change around the cardiac frequency suggests a smaller pulsatile pulmonary blood-volume change compared with surrounding areas, and the region is interpreted as having relatively reduced pulmonary blood flow.

- Regions with a large ΔFPV_L(x, y, frame) appear as deeper red to yellow on PH2-Dynamic/PH2-Summary.

- A large pulsation-related signal change suggests a larger pulsatile pulmonary blood-volume change compared with surrounding areas, and the region is interpreted as having relatively preserved or increased pulmonary blood flow.

- The output PH2-Dynamic and PH2-Summary images are interpreted by physicians together with the static chest radiograph. When no parenchymal abnormality is identified on the static chest radiograph and a region darker than the surrounding area is identified on PH2-Dynamic or PH2-Summary, that region is judged to have a perfusion abnormality.

5 Methods

5.1 Study design

This is a prospective, multicentre, assessor-blinded, case-wise randomised diagnostic performance study designed to verify the superiority of diagnostic accuracy for discriminating CTEPH from non-CTEPH when dynamic chest radiography/pulmonary circulation analysis is added to the standard initial work-up, compared with the standard initial work-up alone.

In this trial, the clinical positioning of the investigational device is as shown in the figure 7; the design aims to evaluate whether the addition of the pulmonary circulation analysis program to the initial work-up improves diagnostic performance for initial discrimination between CTEPH and non-CTEPH.

Standard initial work-up (control) includes:

- Blood tests (complete blood count; D-dimer; total protein; albumin; blood urea nitrogen; creatinine; uric acid; total bilirubin; AST; ALT; LDH; C-reactive protein; sodium; potassium; chloride; estimated glomerular filtration rate; NT-proBNP).

- Static chest radiography (e.g. conventional chest X-ray) (upright posteroanterior view is preferable).

- Resting 12-lead electrocardiogram.

- Pulmonary function tests (TLC measured value and % predicted; VC measured value and % predicted; FEV1 measured value and % predicted; FEV1/FVC%).

- Transthoracic echocardiography (peak tricuspid regurgitation velocity; cine images of parasternal short-axis B-mode and parasternal long-axis and apical four-chamber B-mode and colour Doppler).

Note: The duration of anticoagulant therapy is not restricted.

5.2 Informed consent, screening and registration

Patients in whom pulmonary hypertension is suspected on transthoracic echocardiography may participate in this trial (see Section 3.1). The principal or subinvestigator will judge whether the patient is eligible. The physician will explain trial participation and obtain written informed consent. Initial work-up examinations (including blood tests, static chest radiography, electrocardiography, pulmonary function tests and echocardiography) will be performed within 28 days before registration (screening period). Initial work-up examinations may be performed on the same day as informed consent. After eligibility is confirmed following screening, the patient will be registered. Enrolment will end when 108 patients have been registered.

5.3 Dynamic chest radiography and V/Q scintigraphy

After registration, dynamic chest radiography and V/Q scintigraphy will be performed within 28 days (the interval between the two examinations must be within 14 days).

5.3.1 Standards for acquiring dynamic chest radiography

Dynamic chest radiography (DCR) images are acquired using a stationary digital general-purpose X-ray system capable of pulsed X-ray irradiation and a flat-panel detector–based digital radiography system. Figure 7 illustrates the recommended patient positioning for supine AP acquisition and highlights practical considerations to avoid truncation of the lung fields due to a short source-to-image distance.

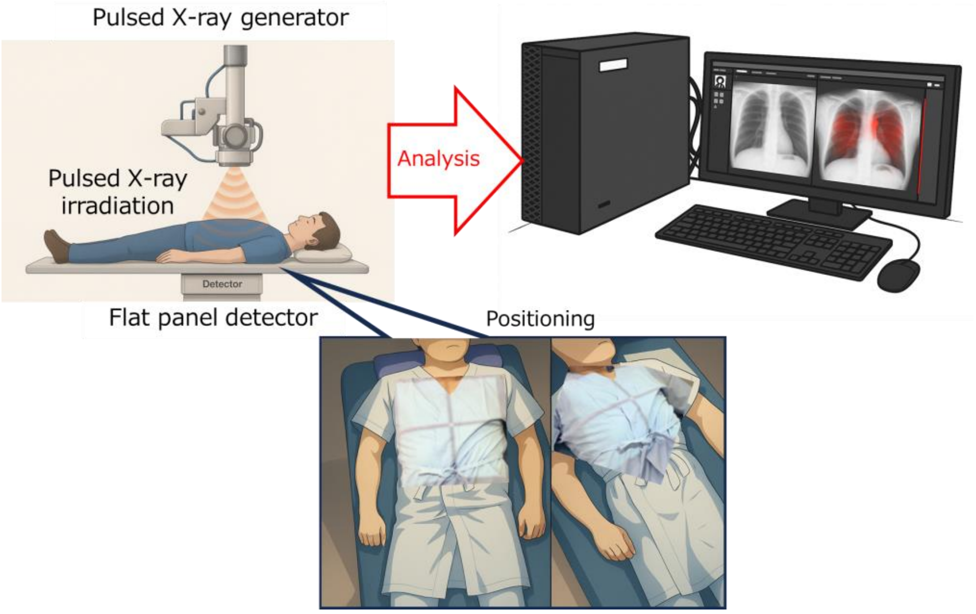

Figure 7. Overview of dynamic chest radiography acquisition and pulmonary circulation analysis.

Dynamic chest radiography is performed using a pulsed X-ray generator and a flat panel detector while the patient holds their breath in the supine anteroposterior position (or upright posteroanterior position where applicable). Sequential X-ray images acquired during pulsed X-ray irradiation are transferred to a dedicated workstation, where the pulmonary circulation analysis program processes the image series to generate dynamic and summary perfusion-surrogate images. Representative examples of patient positioning for dynamic chest radiography are shown.

The steps from image acquisition to generation of pulmonary circulation analysis images are summarised in Table 5. All steps are performed by radiologic technologists.

Step	Description
1)	Acquire a static chest radiograph (CXR) and dynamic chest radiography (DCR). • CXR: X-ray exposure parameters are set according to body mass index (next table). Patient position and projection are aligned with those used for DCR. • DCR: X-ray exposure parameters are set so that the signal-to-noise ratio of the dynamic images is maintained above a prespecified threshold as far as possible irrespective of body mass index. Patient position/projection: supine AP (recommended) or upright PA. Breath-hold: 6–10 s (typically 7 s). Image size: 17 × 17 in (424.8 × 424.8 mm; 1,062 × 1,062 pixels; 400 µm pitch) or 14 × 17 in (348.8 × 425.6 mm; 872 × 1,064 pixels; 400 µm pitch). Signal-value dynamic range: 16-bit (0–65,535). Pulse repetition frequency: 15 Hz for all participants. • Procedure: Position the participant appropriately. When acquiring in the supine position, note that a shorter source-to-image distance may narrow the irradiation field; confirm that the lung fields are not excluded. Confirm that the irradiation field covers the entire panel. Perform a practice breath-hold, then acquire DCR according to the breath-hold protocol.
2)	Visually confirm the acquired CXR and DCR. Exclude inappropriate images and

	repeat acquisition when necessary.<br>Inappropriate image criteria: insufficient dose; body motion; marked rib motion; marked diaphragmatic motion (inadequate breath-hold); failure to secure the background ROI.
3)	Transfer the CXR to the diagnostic viewer and transfer the DCR to the DI-X1 workstation on which PH2-MODE is installed.
4)	On the DI-X1 workstation, use PH2-MODE (both KONICA MINOLTA INC. Tokyo, Japan) to automatically generate the pulmonary circulation analysis movie (PH2-Dynamic) and summary image (PH2-Summary). Confirm the following checkpoints (see the separate “Procedure for creating analysis images” for details): background ROI position; heart ROI position; and lung boundary line.
5)	Visually confirm the pulmonary circulation analysis images and exclude inappropriate images.<br>Inappropriate analysis-image criteria: marked artefacts due to body motion; marked artefacts due to rib motion; marked artefacts due to inadequate breath-hold.
6)	Finalise PH2-Dynamic and PH2-Summary on the DI-X1 workstation and transmit them to the diagnostic viewer.

Recommended pulsed X-ray irradiation conditions according to body mass index (supine AP projection)

BMI category	Tube voltage (kV)	Tube current (mA)	Exposure time (ms)
BMI<17	85	250	2.2
17≤BMI<20	95	250	1.8
20≤BMI<23	95	250	2.5
23≤BMI<26	95	250	3.2
26≤BMI<29	95	250	4
29≤BMI<32	95	250	4.5
32≤BMI	95	250	5

Recommended pulsed X-ray irradiation conditions according to body mass index (upright PA projection)

BMI category	Tube voltage (kV)	Tube current (mA)	Exposure time (ms)
BMI<17	85	80	8.0
17≤BMI<20	100	80	5.0
20≤BMI<23	100	80	6.3
23≤BMI<26	100	80	7.1
26≤BMI<29	110	250	2.2
29≤BMI<32	110	250	2.5
32≤BMI	110	250	3.6

CTEPH discrimination criteria (image interpretation committee): Determined in accordance with the SOP for image interpretation.

Six-region assessment of perfusion defects on pulmonary circulation analysis images (image interpretation committee): For each of the six lung regions, record “present” for regions judged to have embolic-type perfusion abnormalities and “absent” for regions judged not to have embolic-type perfusion abnormalities. Static chest radiography is not used for this six-region assessment (V/Q mismatch is not considered).

5.3.2 Six-region assessment of perfusion defects on perfusion scintigraphy

For perfusion scintigraphy, technetium-99m macroaggregated albumin (99mTc-MAA) will be administered, and lung perfusion images will be acquired using a gamma camera SPECT system or SPECT/CT system. Multi-direction planar images (including anterior and posterior views; two views are acceptable) and SPECT images will be obtained; where available, CT images for fusion will also be acquired.

Six-region assessment (image interpretation committee): Using SPECT/CT (or SPECT when CT is unavailable) to define the cranio-caudal extent of the lungs, each lung will be divided into three equal zones (upper, middle and lower), resulting in six zones in total. A reference anterior planar image with six-zone division lines will be created for scoring. For each of the six zones, readers will record "present" when an embolic-type perfusion abnormality is judged to be present and "absent" when it is judged not to be present. Ventilation scintigraphy is not used for this six-region assessment; therefore, V/Q mismatch is not considered in this analysis.

5.4 CTEPH versus non-CTEPH diagnosis

CTEPH versus non-CTEPH diagnosis will be made by the principal or subinvestigator. To diagnose CTEPH, concordant findings must be present on V/Q scintigraphy, organised thrombi must be identified within the pulmonary arteries on at least one modality (CT pulmonary angiography or catheter-based pulmonary angiography), and pulmonary hypertension must be confirmed by right heart catheterisation. Because this trial is focused on diagnosing pulmonary perfusion impairment, the strict duration of anticoagulant therapy (usually 3 months) is not included as a requirement for diagnosis in this trial. The CTEPH versus non-CTEPH diagnosis will be determined within 42 days after registration.

5.5 End of testing (EoT)

Registered subjects will reach the end of testing (EoT) upon completion of dynamic chest radiography and V/Q scintigraphy. After the principal or subinvestigator determines the diagnosis of CTEPH versus non-CTEPH, all required information will be entered into the electronic data capture (EDC) system without omissions. Adverse events will be collected in relation to dynamic chest radiography. If a serious adverse event occurs, appropriate measures prioritising patient safety will be taken. In the event of discontinuation, the prespecified assessments at discontinuation will be performed as far as possible within 7 days, and the date and reason for discontinuation will be recorded. Serious adverse events will be reported appropriately in accordance with Section 8 (Adverse events and malfunctions).

5.6 Randomisation, end of study (EoS) and image reading

After registration, once the subject reaches EoT and the principal or subinvestigator has determined the diagnosis of CTEPH versus non-CTEPH, the data manager will confirm that there are no missing data in the EDC system. Using an allocation tool, cases will be stratified by the final diagnosis so that the numbers of CTEPH and non-CTEPH cases are balanced between the two reading groups and will then be allocated 1:1 to (i) the initial work-up–only

reading group and (ii) the initial work-up plus pulmonary circulation analysis image reading group. After allocation, the case reaches end of study (EoS). For cases allocated to each group, a reading ID will be assigned so that the group can be identified by the image interpretation committee. Information (initial work-up data) will be sent to image readers, excluding the final diagnosis. For all cases, pulmonary circulation analysis images will be created from dynamic chest radiography acquired by the investigational device (see the appendix “Procedure for creating analysis images from dynamic chest radiography”). Trained readers in the image interpretation committee will interpret the images (see Section 13 Image interpretation committee; the SOP for operating the image interpretation committee; and the SOP for image interpretation). Reading results, including the initial discrimination result of CTEPH versus non-CTEPH by the image interpretation committee, will be sent to the statistical analyst, and the endpoints will be calculated.

The trial will be considered completed when all enrolled cases have reached EoS and all related trial procedures have been completed.

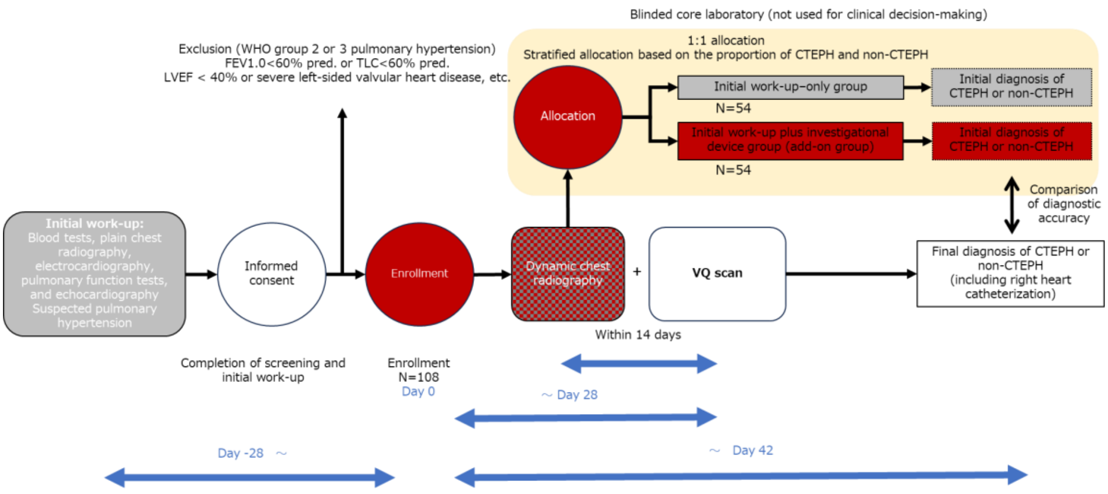

Study flow (primary analysis)

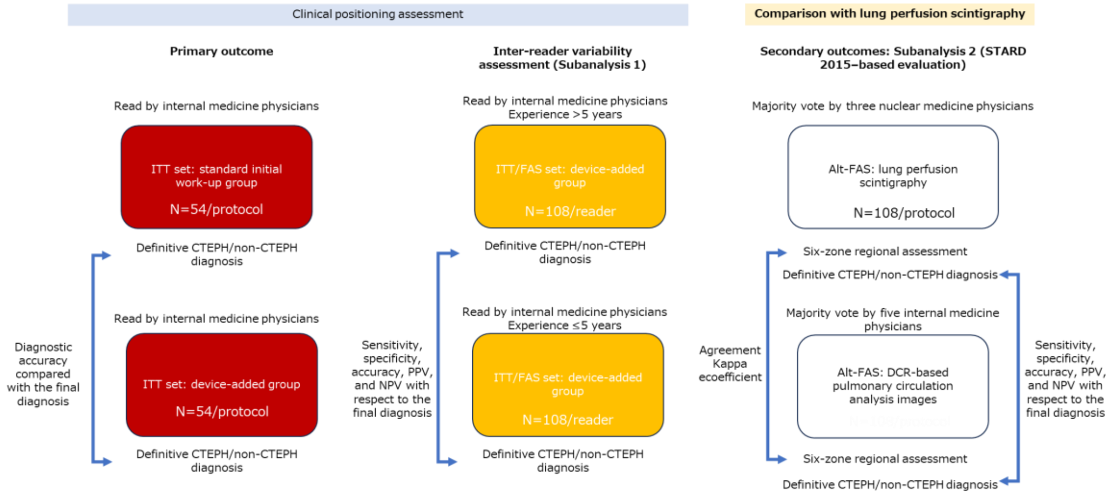

Reader study outline (blinded core laboratory: primary and secondary outcomes, and inter-reader variability assessment)

5.7 Endpoints and rationale

In this trial, image evaluations will be conducted by an independent image interpretation committee. The composition of the independent committee and the method for determining evaluation outcomes are specified in Section 13 (Image interpretation committee).

5.7.1 Primary endpoint

Diagnostic accuracy (Sensitivity, Specificity, positive predictive value and negative predictive value will also be assessed as secondary endpoints) for initial discrimination between CTEPH and non-CTEPH in the intention-to-treat (ITT) population, comparing the group of the standard initial work-up alone versus the device -added group.

5.7.2 Secondary endpoints

- Comparison of sensitivity, specificity, positive predictive value and negative predictive value for initial discrimination between CTEPH and non-CTEPH between the group of the standard initial work-up alone versus the device -added group in the ITT population.

- Agreement (kappa coefficient) between the pulmonary circulation analysis program and perfusion scintigraphy regarding the presence of regional perfusion defects in each of six lung regions.

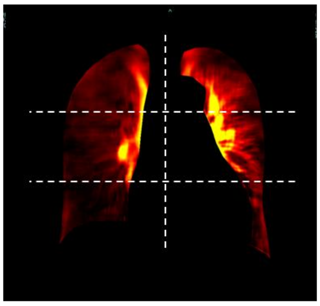

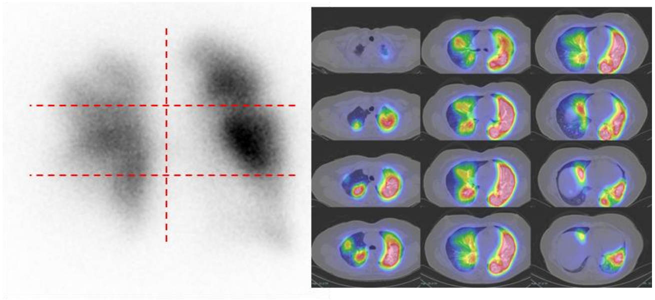

Right	Left
Upper:<br>Perfusion defect<br>(+ / -)	Upper:<br>Perfusion defect<br>(+ / -)
Middle:<br>Perfusion defect<br>(+ / -)	Middle:<br>Perfusion defect<br>(+ / -)
Lower:<br>Perfusion defect<br>(+ / -)	Lower:<br>Perfusion defect<br>(+ / -)

Right	Left
Upper:<br>Segmental perfusion defect<br>(+ / -)	Upper:<br>Segmental perfusion defect<br>(+ / -)
Middle:<br>Segmental perfusion defect<br>(+ / -)	Middle:<br>Segmental perfusion defect<br>(+ / -)
Lower:<br>Segmental perfusion defect<br>(+ / -)	Lower:<br>Segmental perfusion defect<br>(+ / -)

Kappa coefficient for agreement in six area

- Performance evaluation of dynamic chest radiography/pulmonary circulation analysis program for discriminating CTEPH versus non-CTEPH using perfusion scintigraphy as the reference standard: sensitivity, specificity, accuracy, PPV and NPV, in accordance with the STARD 2015 guideline.

5.7.3 Exploratory endpoints

- Subgroup analyses in the primary endpoint according to sex (male vs female): comparison of accuracy, sensitivity and specificity between the group of the standard initial work-up alone versus the device -added group.

- Subgroup analyses in the primary endpoint according to obesity (BMI ≥25 kg/m2 vs BMI <25 kg/m2)[20]: comparison of accuracy, sensitivity and specificity between the group of the standard initial work-up alone versus the device -added group.

- Subgroup analyses in the primary endpoint according to pulmonary vascular resistance (>4.0 Wood units vs ≤4.0 Wood units): comparison of accuracy, sensitivity and specificity between the group of the standard initial work-up alone versus the device -added group.

- Subgroup analyses in the primary endpoint according to NT-proBNP (≥300 pg/mL vs <300 pg/mL): comparison of accuracy, sensitivity and specificity between the group of the standard initial work-up alone versus the device -added group.

- Subgroup analyses in the primary endpoint according to peak tricuspid regurgitation velocity (greater than the median vs ≤ the median): comparison of accuracy, sensitivity and specificity between the group of the standard initial work-up alone versus the device -added group.

- Subgroup analyses in the primary endpoint according to cardiac rhythm (sinus rhythm vs non-sinus rhythm): comparison of accuracy, sensitivity and specificity between the group of the standard initial work-up alone versus the device -added group.

- Subanalysis 1: comparison of accuracy, sensitivity, specificity, PPV and NPV between the control and intervention groups stratified by reader experience (more than 5 years vs 5 years or less) in the ITT population and in the full analysis set (FAS).

- Examination time (time spent in the examination room), radiation dose (dose area product [DAP], only in facilities able to measure it) and number of acquisitions including repeats when acquiring dynamic chest radiography for the pulmonary circulation analysis program in the ITT population and FAS.

- Exclusion proportion of pulmonary circulation analysis images reconstructed from dynamic chest radiography in the ITT population and FAS (including reasons for exclusion, e.g., failure to acquire dynamic chest radiography, failure of analysis by the program, or images judged inappropriate by investigators).

5.7.4 Safety endpoint

Adverse events during dynamic chest radiography and device malfunctions during the trial period.

6 Participant registration

6.1 Registration procedures

The principal or subinvestigator will obtain written informed consent from subjects judged likely to be eligible for this trial. Each consented subject will be assigned a subject identification code, which will be recorded and managed in the subject identification code list. After confirming that the subject meets all inclusion criteria and none of the exclusion criteria, the investigator will register the case using the case registration function of the electronic data capture (EDC) system. If there are questions regarding eligibility criteria, the investigator should confirm with the coordinating investigator.

EDC system: cubeCDMS.

For inquiries regarding the registration procedure and use of the EDC system: Micron, Inc. (TEL: 03-6631-3696; business hours: 9:00-17:00).

For inquiries regarding inclusion/exclusion criteria: Coordinating investigator Kotaro Abe (Department of Cardiovascular Medicine, Kyushu University Hospital; TEL: 092-642-5360; e-mail: abe.kotaro.232@m.kyushu-u.ac.jp).

6.2 Precautions at registration

- Dynamic chest radiography should be performed after registration; however, if dynamic chest radiography has been performed between screening and registration, those images may be used in lieu of post-registration imaging.

- The interval between dynamic chest radiography and perfusion scintigraphy must be within 14 days.

- Registration will not be accepted until eligibility is confirmed based on data entered into the EDC system.

- A registered subject will not have their registration cancelled except in the event of withdrawal of consent that includes refusal to allow use of data for the trial.

- In the event of duplicate registration, the information from the first registration (e.g., registration number) will be adopted.

- If erroneous registration or duplicate registration is identified, the investigator should promptly contact the point of contact described in Section 6.1 (Registration procedures).

7 Assessments and schedule

7.1 Assessment items

7.1.1 Screening assessments (items marked with * are part of the standard initial work-up)

After obtaining informed consent and before registration, the principal or subinvestigator will perform the following assessments, observations and examinations to confirm eligibility.

To minimise subject burden, test results obtained before informed consent may be used if within acceptable time windows.

Subject background

- Sex*; age (at the time of informed consent)*; use of anticoagulants (yes/no), drug name and dosage; medical history (diseases related to exclusion criteria); comorbidities.

- Height, weight, body mass index (BMI; calculated automatically) and body surface area (BSA; calculated automatically).

- World Health Organization (WHO) functional class.*

- Resting 12-lead electrocardiogram* and rhythm classification (sinus rhythm vs non-sinus rhythm). If non-sinus rhythm, document the type of baseline rhythm and heart rate.

- Pulmonary function tests (TLC measured value and % predicted; VC measured value and % predicted; FEV1 measured value and % predicted; FEV1/FVC%).*

- Transthoracic echocardiography.*

- Peak tricuspid regurgitation velocity; LVEF; cine images of parasternal long-axis and short-axis views and apical four-chamber view in B-mode and colour Doppler; and presence/absence of other echocardiographic findings suggestive of pulmonary hypertension (see Tables 3 and 4).

- Static chest radiography (e.g. conventional chest X-ray) (upright PA view is preferable).*

- Blood tests*:

- - Complete blood count: white blood cell count, haemoglobin, platelet count.

- - Biochemistry: total protein, albumin, blood urea nitrogen, creatinine, uric acid, total bilirubin, AST, ALT, LDH, C-reactive protein, sodium, potassium, chloride, estimated glomerular filtration rate.

- - Coagulation test: D-dimer.

- - Hormonal test: NT-proBNP.

- Presence/absence of organised thrombi on contrast-enhanced CT or pulmonary angiography. If performed between 180 days before registration and before the investigator’s diagnosis of CTEPH/non-CTEPH, data from other institutions may be used.

7.1.2 Dynamic chest radiography

After obtaining a static chest radiograph (e.g. conventional chest X-ray) (supine anteroposterior view is recommended; alternatively upright posteroanterior view), dynamic chest radiography is acquired during breath-hold for 6–10 seconds (approximately 105 frames). For details of the imaging procedure, refer to “Section 5.2 Standards for acquiring dynamic chest radiography”.

The body position and projection during imaging and the times of entering and leaving the examination room will be recorded; examination time will be calculated from the time spent in the room. In facilities capable of measurement, radiation dose (DAP) will also be measured. The number of acquisitions including repeats due to inadequate breath-hold, etc., and the reasons for repeats will be recorded for each repeat. If imaging could not be performed for any reason, the reason for inability to acquire images will be recorded.

Dynamic chest radiography images with personal information masked will be submitted to the image interpretation committee.

7.1.3 V/Q scintigraphy / SPECT-CT

For ventilation scintigraphy, krypton-81m gas or technetium-99m aerosol (gas) will be used; for perfusion scintigraphy, technetium-99m macroaggregated human serum albumin (99mTc-MAA) will be used. V/Q scintigraphy is obtained by imaging the lungs with a gamma camera with detectors positioned outside the body.8 The presence/absence of regional ventilation–perfusion mismatch will be evaluated and recorded. Multi-direction planar images (including anterior and posterior views; only two views are acceptable), SPECT images, and (where available) CT images for fusion will be acquired. For details, refer to "section 5.3 Evaluation of pulmonary perfusion impairment in each of six lung regions on perfusion scintigraphy".

Obtained images will have personal information masked and will be submitted to the image interpretation committee.

7.1.4 Right heart catheterisation

Right heart catheterisation will be performed under resting supine conditions to measure mean right atrial pressure, systolic/mean/diastolic pulmonary arterial pressure, mean pulmonary capillary wedge pressure, cardiac output (thermodilution is preferable), cardiac index and pulmonary vascular resistance (Wood units).

7.1.5 Diagnosis

After completion of the above examinations, the principal or subinvestigator will determine the diagnosis of CTEPH or non-CTEPH according to the diagnostic criteria below.

- CTEPH: all of the following are met:

- 1) V/Q scintigraphy: segmental defects or multiple subsegmental defects on perfusion imaging.

- 2) CT pulmonary angiography and/or catheter-based pulmonary angiography: findings of organised thrombi in the pulmonary arteries.

- 3) Right heart catheterisation: pulmonary hypertension, defined as mPAP >20 mmHg at rest and pulmonary vascular resistance ≥3 Wood units.

- Non-CTEPH: does not meet the criteria for CTEPH above.

- a) A definitive diagnosis other than CTEPH is made (pulmonary hypertension clinical classification).

- b) Right heart catheterisation shows mPAP ≤20 mmHg (no pulmonary hypertension).

- c) An appropriate diagnosis cannot be made or appropriate images/data are not available, and the principal/subinvestigator judges the case to be inappropriate for evaluation in this trial.

Note: The definition of pulmonary hypertension is based on the 2022 ESC/ERS Guidelines, where pulmonary hypertension is defined as mPAP >20 mmHg. Therefore, mPAP >20 mmHg and pulmonary vascular resistance ≥3 Wood units are used in the diagnostic criterion in this protocol.[21]

7.1.6 Initial discrimination at the blinded core laboratory

Initial discrimination between CTEPH and non-CTEPH at the blinded core laboratory is not intended to be a definitive diagnosis, but rather to estimate the pre-test probability before specialised examinations. Accordingly, the judgement of whether the findings are suggestive of CTEPH is made comprehensively based on the initial work-up plus the pulmonary circulation analysis program (in the intervention group). If a regional perfusion defect is present on the pulmonary circulation analysis images, the case will be classified as CTEPH even if the initial work-up shows findings suggestive of non-CTEPH (e.g., left ventricular hypertrophy or pulmonary function abnormalities). For details of the reading procedure, refer to the SOP for image interpretation.

7.2 Schedule

The schedule of assessments is shown in Table 5.

Table 5. Study schedule

	Informed consent	Screening	Registration	Examination			Allocation /image reconstruction	End of study (EoS)
Visit window	Day −28 ～ Day 0		Index Day 0	Day 0～ Day 28	Day −28 ～Day 42	Day 0 ～ Day 42		Within 7 days from EoS
Informed consent	○							
Eligibility			○					
Enrollment			○※1					
Patients background		○						
Height/weight		○						

WHO functional class		○						
Blood test		○※2						
ECG		○※2						
Pulmonary function test		○※2						
UCG		○※2						
Chest X-ray		○※2						
Dynamic chest radiography				○※3				
V/Q scintigraphy / SPECT-CT				○※3				
Right heart catherterisation					○※2			
Final CTEPH/non-CTEPH diagnosis by investigator						○※2		○
Adverse events				○				○
Malfunction				○			○	

* Items marked with an asterisk (*) constitute the standard initial work-up information.

※1 Dynamic chest radiography should be performed within 28 days after registration; however, if performed between screening and registration, those images may be used.

※2 Right heart catheterisation should be performed within 28 days after registration; however, if performed within 180 days before registration, those data may be used.

EoT: End of testing; EoS: End of study.

8 Adverse events and malfunctions

8.1 Definition of adverse events

An “adverse event” refers to any unfavourable or unintended disease or disorder and its signs (including abnormal clinical laboratory values) that occurs in subjects, users or other

persons during use of the investigational device, regardless of whether a causal relationship with the investigational device is present. For events occurring in persons other than the subject, only those suspected to be influenced by use of the investigational device are included.

8.2 Evaluation and reporting of adverse events

The intervention in this trial is acquisition of dynamic chest radiography. This is a non-invasive imaging examination with radiation exposure comparable to standard chest radiography (0.1–0.4 mSv) and does not exceed the scope of routine clinical practice. If an adverse event occurs in relation to the dynamic chest radiography procedure, the following will be recorded: adverse event name; the subject in whom it occurred; onset date; outcome; outcome date; severity; seriousness; causal relationship with the investigational device; measures taken regarding the investigational device; and treatments taken for the adverse event (including concomitant drugs and concomitant therapies).

If a serious adverse event occurs, the principal investigator will report it in accordance with Section 8.5 (Reporting procedures for serious adverse events and malfunctions).

1) Definition of serious adverse events

Among adverse events, those that meet any of the following criteria are defined as serious adverse events:

- Death.

- A life-threatening event.

- Event requiring hospitalisation for treatment or prolongation of existing hospitalisation.

- Disability.

- Event that may result in disability.

- Event that is considered serious in accordance with the above items (1–5).

- Congenital disease or abnormality in subsequent generations.

2) Causal relationship between adverse events and the investigational device

Causality between an adverse event and the investigational device will be classified as follows. “Related” refers to an event in which there is at least a reasonable possibility of a causal relationship between the adverse event and the investigational device and a causal relationship cannot be ruled out.

- Unrelated

- Related

3) Severity of adverse events

If an adverse event occurs, severity will be recorded using the following Grade 1–5 scale:

- Grade 1: Mild – no symptoms or mild symptoms; no treatment required.

- Grade 2: Moderate – non-invasive treatment required.

- Grade 3: Severe or medically significant but not immediately life-threatening.

- Grade 4: Life-threatening.

- Grade 5: Death.

8.3 Definition of malfunctions

A "malfunction" refers to any problem with the investigational device related broadly to quality, safety or performance, such as damage or malfunction, regardless of whether it arises at the stage of design, delivery, storage or use.

8.4 Evaluation of malfunctions

For malfunctions that occur during use of the investigational device, the following will be recorded as applicable: malfunction name; date and time of occurrence; situation of the malfunction; contributing factors (if any); whether any harm occurred; severity; seriousness; causal relationship with the investigational device; serial number of the investigational device; whether any action was taken for the malfunction (and details if yes); and whether there is a risk of a serious adverse event due to the malfunction.

Malfunctions related to the investigational device include poor image quality or program operational failures when using the pulmonary circulation analysis program. For malfunctions occurring during the trial period, the malfunction name, date and time of occurrence, situation and contributing factors (if any) will be recorded.

If a malfunction is judged to potentially lead to a serious adverse event as defined in Section 8.2.1, the principal investigator will report it in the same manner as a serious adverse event in accordance with Section 8.5.

8.5 Reporting procedures for serious adverse events and malfunctions

If a serious adverse event occurs in relation to the dynamic chest radiography procedure, or if a serious adverse event is suspected to occur due to a malfunction, the principal investigator will, in accordance with the trial's SOP for handling safety information, report the event to the head of the implementing medical institution and to the coordinating investigator (contact information below), and will also coordinate with the investigational device provider as necessary.

The principal investigator must conduct follow-up investigations for all serious adverse events, and promptly provide the obtained information to the head of the implementing medical institution, the coordinating investigator, principal investigators at other implementing medical institutions, and the investigational device provider.

Contact for serious adverse events

Kyushu University Hospital, ARO Next-Generation Medical Center, Safety Information Management Unit

E-mail: cteph-dcr@aro.kyushu-u.ac.jp (weekdays, nights and holidays)

TEL: +81-92-642-6290 FAX: +81-92-642-6292 (weekdays 08:30–17:00)

The coordinating investigator will determine, in accordance with the SOP for handling safety information, whether a report to the Pharmaceuticals and Medical Devices Agency (PMDA) is required for the event, and will consult with the principal investigator. If reporting is deemed necessary, the coordinating investigator will report to the PMDA in accordance with the SOP.

8.6 Information on quality, effectiveness and malfunctions of the investigational device

The coordinating investigator will collect information on the quality, effectiveness and malfunctions of the investigational device (including domestic and overseas case information, foreign regulatory actions, research reports, etc.) either directly or from the investigational device provider and other sources. The collected information will be handled in accordance with the SOP for handling safety information.

8.7 Periodic safety reporting

The coordinating investigator will conduct periodic reporting in accordance with Article 274-2, Paragraph 4 of the Enforcement Regulations of the Pharmaceuticals and Medical Devices Act, following the SOP for handling safety information, to comprehensively evaluate safety information related to the investigational device.

9 Target sample size and study period

9.1 Planned sample size

A total of 108 cases will be registered (54 cases in the standard initial work-up group and 54 cases in the investigational device group).

Rationale for sample size determination is described in Section 10.1. Based on data from Kyushu University Hospital from January to April 2024, among 17 cases in which pulmonary hypertension was strongly suspected on transthoracic echocardiography and V/Q scintigraphy and right heart catheterisation were performed, the diagnoses were CTEPH in 41% (7/17) and non-CTEPH in 59% (10/17). Assuming a similar 50:50 distribution of CTEPH and non-CTEPH in the planned sample size, at least 10 cases of CTEPH are expected. If fewer than 10 CTEPH cases are included, additional registration will continue until at least 10 CTEPH cases are obtained.

9.2 Planned study period

Planned study period: June 2025 to May 2027.

Planned registration period: June 2025 to February 2027.

10 Statistical considerations

10.1 Rationale for sample size determination

The diagnostic performance of V/Q scintigraphy is extremely high, with sensitivity and specificity reported as 90–100% and 94–100%, respectively.[1,3,7,8] However, access to V/Q scintigraphy may be delayed and it is not available in many non-expert centres; therefore, adding the pulmonary circulation analysis program to the initial work-up is expected to improve early discrimination of CTEPH.

In the retrospective study conducted at Kyushu University Hospital (Section 1.5), the diagnostic performance of dynamic chest radiography/pulmonary circulation analysis for CTEPH discrimination was high (accuracy 0.92), whereas published data indicate limited diagnostic performance of static chest radiography alone.[18,19] Based on these data, the expected accuracy for initial discrimination using the standard initial work-up alone is assumed to be approximately 0.705, and the expected accuracy when adding the pulmonary circulation analysis program is assumed to be approximately 0.92 (difference 0.215).

To detect an accuracy difference of 0.215 between the two groups with a two-sided significance level of 5% and 80% power under a 1:1 allocation, the required sample size is calculated as 102 cases (51 per group). Allowing for approximately 5% dropouts or non-evaluable cases, the target sample size was set at 108 cases (54 per group).

10.2 Analysis populations

10.2.1 Intention-to-treat (ITT) population

The ITT population is defined as all registered subjects excluding the following:

- Cases judged by the principal investigator to have difficulty in making a clear discrimination diagnosis of CTEPH versus non-CTEPH.

- Cases found after registration to not meet inclusion criteria or to meet exclusion criteria.

- Cases with major non-compliance with Good Clinical Practice (GCP).

10.2.2 Full analysis set (FAS)

The FAS is defined as the ITT population excluding the following:

- Cases in which any of the initial work-up tests (blood tests, static chest radiography, 12-lead ECG, or pulmonary function tests) were not performed, or cases with three or more missing items in blood tests.

- Cases without dynamic chest radiography images, or cases in which the image interpretation committee judges that appropriate dynamic chest radiography images required to perform the pulmonary circulation analysis program are not available (e.g., due to inadequate breath-hold or body motion).

10.2.3 Alternative full analysis set for six-region assessment (alt-FAS)

The alt-FAS is defined as the ITT population excluding the following. This set includes all cases with dynamic chest radiography performed, regardless of group allocation, for the six-region assessment analyses.

- Cases without dynamic chest radiography images, or cases in which the image interpretation committee judges that appropriate dynamic chest radiography images required to perform the pulmonary circulation analysis program are not available (e.g., due to inadequate breath-hold or body motion).

- Cases without perfusion scintigraphy images, or cases in which the image interpretation committee judges that appropriate perfusion scintigraphy images are not available.

- Cases in which the interval between dynamic chest radiography acquisition and perfusion scintigraphy is 15 days or longer.

10.2.4 Safety analysis set (SAS)

The SAS is defined as all registered subjects excluding those in whom dynamic chest radiography was not acquired even once.

Analysis populations will be finalised before database lock at a case review meeting. If issues not described here need to be considered for specific cases, the decision will be made at the case review meeting.

10.3 Analysis items and methods

10.3.1 Primary analysis

The primary analysis will be performed in the ITT population. This trial is designed to demonstrate superiority of the intervention. The null hypothesis is that the diagnostic accuracy of initial discrimination in the intervention group is less than or equal to that in the control group; the alternative hypothesis is that the diagnostic accuracy in the intervention group is greater than that in the control group.

A two-sided significance level of 5% will be used. The specific statistical test for the primary endpoint will be described in the statistical analysis plan.

10.3.2 Secondary and exploratory analyses

Secondary and exploratory endpoint analyses will be performed as described in Section 5.7 Endpoints and rationale.

10.3.3 Safety analysis

Adverse events and malfunctions will be summarised in the SAS.

10.3.4 Other analyses

The same analyses as in the ITT population will also be performed in the FAS.

10.4 Handling of missing data

Missing data will not be imputed. Analyses will be conducted based on available data.

10.5 Multiplicity adjustment

No multiplicity adjustment will be performed.

10.6 Interim analysis

No interim analysis is planned.

11 Study completion or termination

11.1 Study completion

After all trial procedures have been completed for all subjects, the principal investigator will submit a written report to the head of the implementing medical institution stating that the trial has been completed and providing an outline of the trial results. The head of the implementing medical institution will promptly notify the institutional review board (IRB) in writing of the completion of the trial, and will report an outline of the trial results based on the report submitted by the principal investigator.

11.2 Study termination

11.2.1 Termination criteria

- If the coordinating investigator judges that the trial should not be continued (e.g., serious adverse events or malfunctions related to the investigational device).

- If ethical or scientific issues are identified in the trial.

- If a termination recommendation is issued by the IRB or the regulatory authority.

11.2.2 Procedures in the event of termination

If any matter corresponding to the termination criteria occurs, the coordinating investigator and the principal investigator will immediately consult and decide whether to terminate the trial. If the decision is made to terminate the trial, the coordinating investigator will, as necessary, communicate the reason for termination and subsequent actions in writing to the head of the implementing medical institution, the IRB and other stakeholders. Appropriate measures will be taken to ensure subject safety and to manage any required follow-up.

12 Data handling

12.1 Creation or modification of case report forms

The principal investigator, subinvestigators or study staff will promptly create or modify case report forms using the EDC system in accordance with the data entry manual. Data recorded in the case report forms that are derived from source documents must not contradict the source documents. If inconsistencies exist between source documents and case report forms, the reason must be explained. The principal investigator must ensure that the data in the case report forms and all other reports are accurate and complete. The principal investigator will confirm the appropriateness of the completed case report forms and provide an electronic signature.

12.2 Definition of the original case report form

The original case report form is defined at each stage of creation and storage of electronic data as follows:

- During the trial, all electronic data entered directly into the EDC system are regarded as the original. The EDC system storing the original data will be appropriately backed up to prepare for disasters, system failures, etc.

- After trial completion, all electronic data for the trial will be transferred from the EDC system to optical recording media, which will be regarded as the original. A duplicate will be created as a copy. Thereafter, all electronic data for this trial stored on the EDC server will be invalidated.

12.3 Source documents

In this trial, source documents include the following:

- Records related to the subject's informed consent and provision of information.

- Materials that serve as the basis for entries in case report forms, such as medical records, nursing records, CT images and X-ray images.

- Records related to investigational device management.

For the following items that are entered only into the case report forms, the case report forms will be regarded as the source documents:

- Medical history, comorbidities, concomitant medications, concomitant therapies, and presence/absence of malfunctions.

- Comments and findings, etc.

12.4 Handling of withdrawal of consent

If a subject withdraws consent, the investigator will confirm whether the subject permits continued use of data that have already been collected. If the subject does not permit use of the data, the collected data will be deleted in accordance with applicable procedures and will be excluded from analysis. Details of the subject's decision will be recorded in the medical record.

13 Image interpretation committee

13.1 Composition

The image interpretation committee will perform independent image evaluations for the purpose of determining the outcomes. Committee members will be selected based on their experience with pulmonary hypertension and CTEPH. In order to maintain independence, physicians performing primary endpoint interpretations will be internal medicine physicians who have not been working full-time at a CTEPH expert centre, which is certified by the

Japanese pulmonary circulation and pulmonary hypertension society, in the past three years.

For interpretation of perfusion scintigraphy images used in the six-region regional perfusion defect assessment, nuclear medicine physicians (or radiologists with expertise in nuclear medicine) who are working at a CTEPH expert centre within the past three years will be selected.

13.2 Determination of CTEPH/non-CTEPH in the primary endpoint

For the primary endpoint, five internal medicine physicians will perform the initial discrimination between CTEPH and non-CTEPH in each reading group (standard initial work-up group and standard initial work-up plus pulmonary circulation analysis program group). The five readers in each group will include three readers with more than five years of experience in CTEPH diagnosis and two readers with five years or less of experience.

For the primary endpoint, the adopted interpretation will not be decided by a simple majority vote. Instead, a prespecified correspondence table will be created in advance such that a single reader's judgement is uniquely adopted for each case. Readers will not be informed of which cases constitute the primary endpoint analysis set.

13.3 CTEPH/non-CTEPH determination in the secondary endpoint (pulmonary circulation analysis program images)

For one secondary endpoint, the same five readers as in the primary endpoint will interpret dynamic chest radiography/pulmonary circulation analysis images in the intervention group to determine CTEPH/non-CTEPH. In this evaluation, the determination will be made by majority vote among the five readers.

13.4 Determination of regional perfusion defects (six-region assessment)

For the six-region assessment, each lung is divided into upper, middle and lower regions (six regions in total). Each region will be assessed for the presence or absence of a perfusion defect on pulmonary circulation analysis images and on perfusion scintigraphy images.

Pulmonary circulation analysis images will be interpreted by the same five internal medicine physicians as in the intervention group. Perfusion scintigraphy images will be interpreted by three nuclear medicine physicians from CTEPH expert centres. All designated readers will interpret all cases. The final presence/absence of a regional perfusion defect for each region will be determined by majority vote.

13.5 Reader training

Prior to the start of image interpretation, readers will undergo training on how to interpret pulmonary circulation analysis images. After training, each reader will perform a test interpretation to confirm that the reader has acquired the necessary interpretation skills. The committee will evaluate inter-reader variability of pulmonary circulation analysis images.

14 Record retention

14.1 Retention of essential documents by implementing medical institutions

Implementing medical institutions will retain essential documents related to the trial until the later of: (i) the date of marketing approval for the application for approval of the investigational device in which this trial data are used, or three years after notification of termination of development if development is discontinued; and (ii) three years after trial completion or trial termination.

The head of each implementing medical institution will appoint a person responsible for record retention to retain the documents and will take all necessary measures to prevent loss or damage. The documents must be available for inspection upon request.

14.2 Retention by the institutional review board (IRB)

The IRB of each implementing medical institution will retain essential documents related to the trial until the later of: (i) the date of marketing approval for the application for approval of the investigational device in which this trial data are used, or three years after notification of termination of development if development is discontinued; and (ii) three years after trial completion or trial termination.

14.3 Retention by the coordinating investigator

The coordinating investigator will retain essential documents related to the trial until the later of: (i) five years after the date of marketing approval for the application for approval of the investigational device in which this trial data are used, or three years after notification of termination of development if development is discontinued; and (ii) three years after trial completion or trial termination.

The coordinating investigator will promptly notify principal investigators at implementing medical institutions when marketing approval has been obtained and/or when the retention period has elapsed.

15 Quality control and quality assurance

15.1 Quality control

The coordinating investigator, principal investigators, subinvestigators and study staff will conduct quality control activities to ensure that the trial is conducted appropriately and that the data are reliable, in accordance with applicable laws and regulations and the trial SOPs.

15.2 Monitoring

Monitoring will be conducted in accordance with the Monitoring SOP. Monitoring will include on-site and/or off-site procedures, as appropriate, to confirm that the trial is conducted safely and in compliance with the protocol, applicable regulations, and Good Clinical Practice for medical device trials, and that the data recorded are accurate and complete.

15.3 Audit

Audits are planned to be conducted at least once annually. At the time of protocol Version 3.2, a detailed Audit SOP has not yet been finalised; however, audits will be performed by personnel independent of the conduct and monitoring of the trial, and will assess compliance with the protocol and applicable regulatory requirements. The Audit SOP will be prepared before the first audit is conducted.

15.4 Direct access

The principal investigator will permit direct access to source documents and other trial-related records by monitors, auditors and regulatory authorities as required, while ensuring protection of personal information.

16 Ethical considerations

16.1 Applicable ethical principles and regulations

This trial will be conducted in accordance with the Declaration of Helsinki (revised October 2013), the Pharmaceuticals and Medical Devices Act and related regulations, the Ministerial Ordinance on Good Clinical Practice for Medical Devices (GCP), and the Ethical Guidelines for Medical and Biological Research Involving Human Subjects, as well as other applicable laws and institutional regulations.

16.2 Institutional review board (IRB)

Prior to trial initiation at each implementing medical institution, the protocol, informed consent documents and other relevant materials will be reviewed and approved by the IRB. The IRB will conduct continuing review as required. If new information becomes available that may affect subject safety or willingness to continue participation, the principal investigator will promptly report such information to the head of the implementing medical institution and to the IRB.

16.3 Protection of personal information

The principal investigator will take appropriate measures to protect personal information of subjects in accordance with applicable laws and institutional policies. In particular:

- Subjects will be managed using subject identification codes, and personal identifiers will not be included in data provided outside the implementing medical institution.

- Personal information will be stored in a manner that prevents unauthorised access, leakage, loss or damage.

- When images and data are transferred to the image interpretation committee or other parties, personal information will be masked and de-identified.

16.4 Protocol compliance and amendments

The principal investigator and subinvestigators will conduct the trial in compliance with this protocol. If amendments to the protocol are necessary, the coordinating investigator will

prepare a revised protocol and obtain approval from the head of the implementing medical institution and the IRB as required before implementation.

16.5 Protocol deviations

The principal investigator must not deviate from the protocol without prior approval of the head of the implementing medical institution and the IRB, except when necessary to eliminate an immediate hazard to subjects. Any deviations will be documented, and the reasons and details will be reported to the head of the implementing medical institution and the IRB.

17 Informed consent

17.1 Explanation to subjects

The principal investigator or subinvestigator will explain the following items to the subject using the informed consent document, and will obtain written informed consent before the subject participates in the trial:

- The purpose of the trial.

- The expected duration of participation.

- The trial procedures and the types of examinations to be performed.

- That the trial involves additional imaging (dynamic chest radiography) and associated radiation exposure.

- The reasonably foreseeable risks and inconveniences.

- The expected benefits to the subject and/or others.

- Alternative procedures or treatments that might be available.

- That participation is voluntary and the subject may withdraw consent at any time without disadvantage.

- How confidentiality and protection of personal information will be ensured.

- How trial-related injury will be handled and what compensation is available.

- Any costs to the subject and any reimbursements (if applicable).

- Contact information for questions about the trial and about subject rights.

- That the results may be published and that personal identity will not be disclosed.

- That source documents may be directly accessed by monitors, auditors and regulatory authorities as required.

- That new information that may affect willingness to continue will be provided.

- Any other information required by applicable regulations and institutional policies.

17.2 Obtaining consent

After sufficient explanation, if the subject (or legally acceptable representative, if applicable) agrees to participate, the subject will sign and date the informed consent form. The person obtaining consent will also sign and date the form. A copy of the signed informed consent form will be provided to the subject, and the original will be retained at the implementing medical institution.

17.3 Re-consent

If new information becomes available during the trial that may affect a subject's willingness to continue participation, the subject will be informed promptly. If necessary, revised informed consent documents will be prepared and approved by the IRB, and re-consent will be obtained.

18 Conflict of interest

All personnel involved in this trial will disclose required information to the conflict of interest committee established at their institution in accordance with institutional regulations, and will obtain review and approval.

19 Trial costs and compensation

19.1 Funding source

This trial will be conducted with support from KONICA MINOLTA, INC. The trial is funded by a grant from the Japan Agency for Medical Research and Development (AMED) and by research funding provided by KONICA MINOLTA, INC. to Kyushu University.

19.2 Trial-related costs

Costs for examinations and tests that are normally performed as part of routine clinical care will be handled as part of usual medical expenses covered by health insurance. Any additional examinations required solely for this trial, including dynamic chest radiography, will be handled in accordance with the regulations of each implementing medical institution. Details of cost handling will follow the institutional rules and will be explained to subjects in the informed consent process.

19.3 Compensation for health injury

Because the intervention in this trial is dynamic chest radiography, which is a non-invasive imaging examination with radiation exposure comparable to standard chest radiography, the risk of health injury is considered minimal. Nevertheless, if a subject suffers health injury that is judged to be related to participation in this trial, appropriate medical care and compensation will be provided in accordance with the relevant laws, regulations, and institutional policies. Insurance may be arranged as necessary.

19.4 Reimbursement / burden reduction fee

Payment of any burden reduction fee (reimbursement) to subjects will be made in accordance with the regulations of each implementing medical institution.

20 Dissemination of results

Results obtained in this trial will belong to Kyushu University Hospital. Information on this trial will be registered and disclosed in clinical trial databases such as the Japan Registry of Clinical Trials (jRCT) and ClinicalTrials.gov prior to trial initiation.

Academic presentations related to this trial may be made by the coordinating investigator or by persons who have obtained approval from the coordinating investigator.

21 Trial organisation

The trial organisation is described in the appendix (Appendix 20.1: Trial organisation).

22 References and resources

1. Galie N, Humbert M, Vachiery JL, et al. 2015 ESC/ERS Guidelines for the diagnosis and treatment of pulmonary hypertension: The Joint Task Force for the Diagnosis and Treatment of Pulmonary Hypertension of the European Society of Cardiology (ESC) and the European Respiratory Society (ERS): Endorsed by: Association for European Paediatric and Congenital Cardiology (AEPC), International Society for Heart and Lung Transplantation (ISHLT). Eur Heart J 2016; 37:67-119.
2. 日本循環器学会：肺高血圧症治療ガイドライン（2017 年改訂版）
3. 日本肺高血圧・肺循環学会：慢性血栓塞栓性肺高血圧症（CTEPH）診療ガイドライン（2022 年版）
4. 公益財団法人　難病医学研究財団　難病情報センター　慢性血栓塞栓性肺高血圧症（指定難病 88）特定医療費（指定難病）受給者所持者数年度報告書
5. J Lewczuk, P Piszko, J Jagas, A Porada, S Wójciak, B Sobkowicz, K Wrabec. Prognostic factors in medically treated patients with chronic pulmonary embolism. Chest. 2001;119(3):818-23.
6. Kinoshita H, Aoki T, Motoki H, Wakita T, Onishi Y, Watanabe-Fujinuma E, Kuwahara K. Patient Journey and Disease-Related Burden in Japanese Patients With Chronic Thromboembolic Pulmonary Hypertension: A Mixed Methods Study. Value Health Reg Issues. 2021;24:17-23.
7. Tunariu N, Gibbs SJ, Win Z, Gin-Sing W, Graham A, Gishen P, Al-Nahhas A. Ventilation-perfusion scintigraphy is more sensitive than multidetector CTPA in detecting chronic thromboembolic pulmonary disease as a treatable cause of pulmonary hypertension. Nucl Med 2007; 48: 680–684.
8. Bajc M, Schümichen C, Grüning T, Lindqvist A, Le Roux PY, Alatri A, Bauer RW, Dilic M, Neilly B, Verberne HJ, Delgado Bolton RC, Jonson B. EANM guideline for ventilation/perfusion single-photon emission computed tomography (SPECT) for

diagnosis of pulmonary embolism and beyond. Eur J Nucl Med Mol Imaging. 2019; 46(12):2429-2451.
9. （公社）日本アイソトープ協会医学・薬学部会　全国核医学診療実態調査専門委員会第 8 回全国核医学診療実態調査報告書. RADIOISOTOPES. 2018; 67, 339–387.
10. 自治財政局準公営企業室「公立病院の現状について」に関する報告書 2019. https://www.soumu.go.jp/main_content/000742388.pdf
11. Lasch F, Karch A, Koch A, Derlin T, Voskrebenzev A, Alsady TM, Hoeper MM, Gall H, Roller F, Harth S, Steiner D, Krombach G, Ghofrani HA, Rengier F, Heußel CP, Grünig E, Beitzke D, Hacker M, Lang IM, Behr J, Bartenstein P, Dinkel J, Schmidt KH, Kreitner KF, Frauenfelder T, Ulrich S, Hamer OW, Pfeifer M, Johns CS, Kiely DG, Swift AJ, Wild J, Vogel-Claussen J. Comparison of MRI and VQ-SPECT as a Screening Test for Patients With Suspected CTEPH: CHANGE-MRI Study Design and Rationale. Front Cardiovasc Med. 2020; 9;7:51.
12. Rajaram S, Swift AJ, Telfer A, Hurdman J, Marshall H, Lorenz E, et al. 3D contrast-enhanced lung perfusion MRI is an effective screening tool for chronic thromboembolic pulmonary hypertension: results from the ASPIRE Registry. Thorax. 2013; 68:677–678.
13. Tanaka R: Dynamic chest radiography: flat-panel detector (FPD) based functional X-ray imaging. Radiol Phys Technol. 2016; 9(2):139-53.
14. Tanaka R, Tani T, Nitta N, Tabata T, Matsutani N, Muraoka S, Yoneyama T, Sanada S. Detection of Pulmonary Embolism Based on Reduced Changes in Radiographic Lung Density During Cardiac Beating Using Dynamic Flat-panel Detector: An Animal-based Study. Acad Radiol. 2019; 26(10):1301-1308.
15. Miyatake H, Tabata T, Tsujita Y, Fujino K, Tanaka R, Eguchi Y. Detection of Pulmonary Embolism using a Novel Dynamic Flat-panel Detector System in Monkeys. Cir J. 2021; 85(4)361-368.
16. Yamasaki Y, Kamitani T, Abe K, Hosokawa K, Sagiyama K, Hida T, Matsuura Y, Kitamura Y, Maruoka Y, Isoda T, Baba S, Yoshikawa H, Kuramoto T, Yabuuchi H, Ishigami K. Diagnosis of Pulmonary Hypertension Using Dynamic Chest Radiography. American journal of respiratory and critical care medicine 2021; 204(11):1336-1337.
17. Yamasaki Y, Abe K, Hosokawa K, Kamitani T. A Novel Pulmonary Circulation Imaging using Dynamic Digital Radiography for Chronic Thromboembolic Pulmonary Hypertension.　Eur Heart J. 2020; 41(26):2506.
18. Yamasaki Y, Abe K, KamitaniT, Hosokawa K, Hida T, Sagiyama K, Matsuura Y, Baba S, Isoda T, Maruoka Y, Kitamura Y, Moriyama S, Yoshikawa H, Fukumoto T, Yabuuchi H, Ishigami K. Efficacy of dynamic chest radiography for chronic thromboembolic pulmonary hypertension. Radiology 2023; 306(3):e220908.
19. Takakura K, Yamasaki Y, Kuramoto T, et al. Refined scan protocol for the evaluation of pulmonary perfusion standardized image quality and reduced radiation dose in dynamic chest radiography. Journal of Applied Clinical Medical Physics. 2023
20. 日本肥満学会：新しい肥満の判定と肥満症の診断基準（2020 年版）
21. 2022 ESC/ERS Guidelines for the diagnosis and treatment of pulmonary hypertension. Marc Humbert, Gabor Kovacs, Marius M. Hoeper, Roberto Badagliacca, Rolf M.F. Berger, Margarita Brida, Jørn Carlsen, Andrew J.S. Coats, Pilar Escribano-Subias, Pisana Ferrari, Diogenes S. Ferreira, Hossein Ardeschir Ghofrani, George Giannakoulas, David G. Kiely, Eckhard Mayer, Gergely Meszaros, Blin Nagavci, Karen M. Olsson, Joanna

Pepke-Zaba, Jennifer K. Quint, Göran Rådegran, Gerald Simonneau, Olivier Sitbon, Thomy Tonia, Mark Toshner, Jean-Luc Vachiery, Anton Vonk Noordegraaf, Marion Delcroix, Stephan Rosenkranz, the ESC/ERS Scientific Document Group. European Respiratory Journal Jan 2022, 2200879
