# Supplementary material for: Facilitating early diagnosis of chronic thromboembolic pulmonary hypertension with dynamic chest radiography: Protocol for a multicenter, assessor-blinded, case-wise randomized superiority reader study (FIND-DCR)
Source: PLoS One. 2026 Jun 11;21(6):e0350858. doi: 10.1371/journal.pone.0350858 (PMC13258118; doi:10.1371/journal.pone.0350858)
Supplement: Attachment — Submitted filename: Response to Reviewers.docx [file pone.0350858.s005.docx]

Response to Reviewers

Manuscript ID: PONE-D-25-69091

Title: Facilitating early diagnosis of chronic thromboembolic pulmonary hypertension with dynamic chest radiography: protocol for a multicenter, assessor-blinded, case-wise randomized superiority reader study (FIND-DCR)

**Dear Dr. Taniyama and Reviewer,**

**We sincerely thank you for your careful evaluation of our manuscript and for the constructive comments. We have revised the manuscript to improve clarity, methodological transparency, and compliance with PLOS ONE requirements.**

**All changes are shown in the revised manuscript with tracked changes. Our point-by-point responses are provided below.**

--------------------------------------------------

Response to Journal Requirements

--------------------------------------------------

1. Please ensure that your manuscript meets PLOS ONE's style requirements, including those for file naming. The PLOS ONE style templates can be found at

<https://journals.plos.org/plosone/s/file?id=wjVg/PLOSOne_formatting_sample_main_body.pdf> and

[https://journals.plos.org/plosone/s/file?id=ba62/PLOSOne_formatting_sample_title_authors_affiliations.pdf](https://track.editorialmanager.com/CL0/https:%2F%2Fjournals.plos.org%2Fplosone%2Fs%2Ffile%3Fid=ba62%2FPLOSOne_formatting_sample_title_authors_affiliations.pdf/1/010f019d60d95cd6-e3fc9597-d5ce-449d-8de9-d7edaa28c870-000000/gN8r_6ewsigfKfYKUJnuy_X8KKwQAv6NgtPRa5enPOQ=257)

**Response:**

**We have revised the manuscript files and formatting to align with PLOS ONE style requirements, including file naming and manuscript structure.**

2. Thank you for stating the following financial disclosure:

 “This work is supported by the Japan Agency for Medical Research and Development (AMED), Project for Medical Device and Healthcare, grant number 25hk0102106h0001, and by a research grant from KONICA MINOLTA, INC.”

Please state what role the funders took in the study.  If the funders had no role, please state: "The funders had no role in study design, data collection and analysis, decision to publish, or preparation of the manuscript."

If this statement is not correct you must amend it as needed.

Please include this amended Role of Funder statement in your cover letter; we will change the online submission form on your behalf.

**Response:**

**We appreciate this important request. We have updated the funding information in the cover letter and online submission form to clarify the role of the funders. The revised wording is as follows:**

**Changes made in manuscript:**

**“This work was supported by the Japan Agency for Medical Research and Development (AMED), Project for Medical Device and Healthcare (grant number JP25hk0102106), and by a research grant from KONICA MINOLTA, INC. KONICA MINOLTA, INC. also provided technical support for optimization of the pulmonary circulation analysis program used in the study and for reader training. The funders had no role in study design, data collection and analysis, decision to publish, or preparation of the manuscript.”**

3. Thank you for stating the following in the Competing Interests section:

“KH, YY, KI, and KA have received research funding from KONICA MINOLTA, INC. outside the submitted work. All other authors declare no competing interests.”

We note that one or more of the authors are employed by a commercial company: KONICA MINOLTA, INC.

1. Please provide an amended Funding Statement declaring this commercial affiliation, as well as a statement regarding the Role of Funders in your study. If the funding organization did not play a role in the study design, data collection and analysis, decision to publish, or preparation of the manuscript and only provided financial support in the form of authors' salaries and/or research materials, please review your statements relating to the author contributions, and ensure you have specifically and accurately indicated the role(s) that these authors had in your study. You can update author roles in the Author Contributions section of the online submission form.

Please also include the following statement within your amended Funding Statement.

“The funder provided support in the form of salaries for authors [insert relevant initials], but did not have any additional role in the study design, data collection and analysis, decision to publish, or preparation of the manuscript. The specific roles of these authors are articulated in the ‘author contributions’ section.”

If your commercial affiliation did play a role in your study, please state and explain this role within your updated Funding Statement.

2. Please also provide an updated Competing Interests Statement declaring this commercial affiliation along with any other relevant declarations relating to employment, consultancy, patents, products in development, or marketed products, etc.

Within your Competing Interests Statement, please confirm that this commercial affiliation does not alter your adherence to all PLOS ONE policies on sharing data and materials by including the following statement: "This does not alter our adherence to  PLOS ONE policies on sharing data and materials.” (as detailed online in our guide for authors <http://journals.plos.org/plosone/s/competing-interests>) . If this adherence statement is not accurate and  there are restrictions on sharing of data and/or materials, please state these. Please note that we cannot proceed with consideration of your article until this information has been declared.

Please include both an updated Funding Statement and Competing Interests Statement in your cover letter. We will change the online submission form on your behalf.

**Response:**

**None of the authors have an employment relationship with KONICA MINOLTA INC. We have stated the Funding Statement and Competing Interests Statement for the cover letter and online submission form to clarify the relationship with KONICA MINOLTA, INC. and to state the role of the company more explicitly.**

**Changes made in manuscript:**

**ORIGINAL**

**Page 20, Competing interests**

**KH, YY, KI, and KA have received research funding from KONICA MINOLTA, INC. outside the submitted work. All other authors declare no competing interests.**

**REVISED**

**Page 20, Competing interests**

**“This study received research support from KONICA MINOLTA, INC. KH, YY, KI, and KA have also received research funding from KONICA MINOLTA, INC. outside the submitted work. KONICA MINOLTA, INC. provided technical support for optimization of the pulmonary circulation analysis program used in the study and for reader training. This does not alter our adherence to PLOS ONE policies on sharing data and materials. All other authors declare no competing interests.”**

4. Please note that funding information should not appear in any section or other areas of your manuscript. We will only publish funding information present in the Funding Statement section of the online submission form. Please remove any funding-related text from the manuscript.

**Response:**

**We have removed funding-related text from the manuscript body in accordance with PLOS ONE policy. Funding information is now confined to the Funding Statement in the submission system and cover letter.**

5. We note that your Data Availability Statement is currently as follows: “No datasets were generated or analysed during the current study.
All relevant deidentified data from this study will be made available upon study completion and publication, subject to applicable regulatory requirements and institutional review board approval.”

Please confirm at this time whether or not your submission contains all raw data required to replicate the results of your study. Authors must share the “minimal data set” for their submission. PLOS defines the minimal data set to consist of the data required to replicate all study findings reported in the article, as well as related metadata and methods (<https://journals.plos.org/plosone/s/data-availability#loc-minimal-data-set-definition>).

For example, authors should submit the following data:

- The values behind the means, standard deviations and other measures reported;

- The values used to build graphs;

- The points extracted from images for analysis.

Authors do not need to submit their entire data set if only a portion of the data was used in the reported study.

If your submission does not contain these data, please either upload them as Supporting Information files or deposit them to a stable, public repository and provide us with the relevant URLs, DOIs, or accession numbers. For a list of recommended repositories, please see <https://journals.plos.org/plosone/s/recommended-repositories>.

If there are ethical or legal restrictions on sharing a de-identified data set, please explain them in detail (e.g., data contain potentially sensitive information, data are owned by a third-party organization, etc.) and who has imposed them (e.g., an ethics committee). Please also provide contact information for a data access committee, ethics committee, or other institutional body to which data requests may be sent. If data are owned by a third party, please indicate how others may request data access.

**Response:**

**Thank you for this important clarification. Because the present article is a study protocol and does not report study outcome data, no participant-level dataset underlies the findings reported in this manuscript. To avoid ambiguity, we revised the Data Availability Statement to make this explicit.**

**Changes made in manuscript:**

**ORIGINAL**

**Page 19, Data availability statement**

**“No datasets were generated or analyzed during the current study. All relevant data from this study will be made available upon study completion, in accordance with applicable regulations and institutional policies.”**

**REVISED**

**Page 19, Data availability statement**

**“This manuscript describes a study protocol and does not report study outcome data. Therefore, no participant-level dataset was generated or analyzed for the findings reported in this article. De-identified minimal datasets underlying future reports of study results will be made available at the time of publication of those results in accordance with journal policy and applicable ethical and regulatory requirements.”**

6. When completing the data availability statement of the submission form, you indicated that you will make your data available on acceptance. We strongly recommend all authors decide on a data sharing plan before acceptance, as the process can be lengthy and hold up publication timelines. Please note that, though access restrictions are acceptable now, your entire data will need to be made freely accessible if your manuscript is accepted for publication. This policy applies to all data except where public deposition would breach compliance with the protocol approved by your research ethics board. If you are unable to adhere to our open data policy, please kindly revise your statement to explain your reasoning and we will seek the editor's input on an exemption. Please be assured that, once you have provided your new statement, the assessment of your exemption will not hold up the peer review process.

**Response:**

**We agree and have clarified the data availability plan as above. Because this manuscript is a protocol paper without reported study outcomes, there are no study-result datasets to deposit for the present submission.**

7. Your ethics statement should only appear in the Methods section of your manuscript. If your ethics statement is written in any section besides the Methods, please move it to the Methods section and delete it from any other section. Please ensure that your ethics statement is included in your manuscript, as the ethics statement entered into the online submission form will not be published alongside your manuscript.

**Response:**

**We have moved the ethics approval and informed consent statement into the Methods section and removed duplicative ethics text from other sections of the manuscript, in accordance with journal requirements.**

8. Please upload a new copy of Figure 1, 4 and 5 as the detail is not clear. Please follow the link for more information:  [https://journals.plos.org/plosone/s/figures](https://track.editorialmanager.com/CL0/https:%2F%2Fjournals.plos.org%2Fplosone%2Fs%2Ffigures/1/010f019d60d95cd6-e3fc9597-d5ce-449d-8de9-d7edaa28c870-000000/Jtqy50apDZfHVbRt1e07MURS2YrY2DSD0UuWq-bYapk=257).

**Response:**

**We have prepared and uploaded higher-resolution versions of Figures 1, 4, and 5 so that all details are clearly legible.**

9. Please include captions for your Supporting Information files at the end of your manuscript, and update any in-text citations to match accordingly. Please see our Supporting Information guidelines for more information: [http://journals.plos.org/plosone/s/supporting-information](http://track.editorialmanager.com/CL0/http:%2F%2Fjournals.plos.org%2Fplosone%2Fs%2Fsupporting-information/1/010f019d60d95cd6-e3fc9597-d5ce-449d-8de9-d7edaa28c870-000000/v-KnBlMy-ENDiPnjwhH5hba-ZKQoEfTvmeZ-AE317lg=257).

**Response:**

**We have added captions for all Supporting Information files at the end of the manuscript and revised the in-text citations accordingly.**

10. Thank you for providing your underlying data as Supporting Information.

We note that the data set contains text or data that is not in English. Please note that PLOS is an English-language publisher, so we require data sets to be provided in English as well. Please upload an English-language version of your data set.

This will also allow us to determine if your data follows PLOS standards per our Data Availability policy here: [https://journals.plos.org/plosone/s/data-availability](https://track.editorialmanager.com/CL0/https:%2F%2Fjournals.plos.org%2Fplosone%2Fs%2Fdata-availability/1/010f019d60d95cd6-e3fc9597-d5ce-449d-8de9-d7edaa28c870-000000/OTLt3vpFEkduE4XYan81sg_jGFqdnPOzurwcpdP_b4Q=257).

**Response:**

**We have revised the Supporting Information so that all retained files are provided in English. Specifically, we have not resubmitted the original Japanese-language protocol as Supporting Information and have instead submitted the English version only. Because this manuscript is a study protocol and the revised submission does not rely on any non-English underlying dataset, no non-English dataset remains in the Supporting Information. Therefore, this issue has been addressed.**

11. We note that there is identifying data in the Supporting Information file “IRB_approval_EN.docx” Due to the inclusion of these potentially identifying data, we have removed this file from your file inventory. Prior to sharing human research participant data, authors should consult with an ethics committee to ensure data are shared in accordance with participant consent and all applicable local laws.

Data sharing should never compromise participant privacy. It is therefore not appropriate to publicly share personally identifiable data on human research participants. The following are examples of data that should not be shared:

-Name, initials, physical address

-Ages more specific than whole numbers

-Internet protocol (IP) address

-Specific dates (birth dates, death dates, examination dates, etc.)

-Contact information such as phone number or email address

-Location data

-ID numbers that seem specific (long numbers, include initials, titled “Hospital ID”) rather than random (small numbers in numerical order)

Data that are not directly identifying may also be inappropriate to share, as in combination they can become identifying. For example, data collected from a small group of participants, vulnerable populations, or private groups should not be shared if they involve indirect identifiers (such as sex, ethnicity, location, etc.) that may risk the identification of study participants.

Additional guidance on preparing raw data for publication can be found in our Data Policy (<https://journals.plos.org/plosone/s/data-availability#loc-human-research-participant-data-and-other-sensitive-data>) and in the following article: <http://www.bmj.com/content/340/bmj.c181.long>.

Please remove or anonymize all personal information (<specific identifying information in file to be removed>), ensure that the data shared are in accordance with participant consent, and re-upload a fully anonymized data set. Please note that spreadsheet columns with personal information must be removed and not hidden as all hidden columns will appear in the published file.

**Response:**

**Thank you for this important comment. In response, we have not resubmitted any ethics approval document containing unmasked identifying information as public Supporting Information. The original Japanese IRB/research ethics committee approval letters have been anonymized by masking personal identifying information before submission, in accordance with the journal’s privacy requirements. English translations of the ethics approval documents have also been prepared and submitted separately as requested. We believe that this revision addresses the concern regarding identifying information.**

12. If the reviewer comments include a recommendation to cite specific previously published works, please review and evaluate these publications to determine whether they are relevant and should be cited. There is no requirement to cite these works unless the editor has indicated otherwise.

**Response:**

**We have reviewed all citation-related comments and evaluated relevance carefully. We have revised the reference list only where appropriate and scientifically relevant.**

13. Please review your reference list to ensure that it is complete and correct. If you have cited papers that have been retracted, please include the rationale for doing so in the manuscript text, or remove these references and replace them with relevant current references. Any changes to the reference list should be mentioned in the rebuttal letter that accompanies your revised manuscript. If you need to cite a retracted article, indicate the article’s retracted status in the References list and also include a citation and full reference for the retraction notice.

**Response:**

**We rechecked the reference list for completeness, accuracy, and current relevance and confirmed that no retracted references are cited. We corrected and updated the reference list where appropriate, and all corresponding changes are reflected in the revised manuscript and, where relevant, in the responses below.**

--------------------------------------------------

Response to Additional Editor Comments

--------------------------------------------------

Editor comment 1:

1. What specific mechanistic advantages does DCR-based pulmonary circulation analysis provide over the standard initial work-up in distinguishing CTEPH from non-CTEPH?

**Response:**

**We thank the Editor for this important question. We agree that the mechanistic rationale should be made more explicit. We have revised the Introduction and Discussion to clarify that the standard initial work-up (blood tests, chest radiography, ECG, pulmonary function tests, and echocardiography) provides indirect and often non-specific evidence of pulmonary hypertension and its potential causes, whereas DCR-based pulmonary circulation analysis adds direct dynamic regional perfusion-surrogate information derived from cardiac-cycle-related changes in X-ray translucency during a short breath-hold, without contrast media or radionuclides.**

**This distinction is mechanistically relevant because CTEPH is characterized by embolic-type regional perfusion abnormalities. Prior retrospective work showed that DCR and V/Q scanning had similar performance for detecting CTEPH in patients with pulmonary hypertension (AUC 0.92 vs 0.93, with substantial agreement, κ = 0.79), supporting the biological plausibility that DCR can reveal perfusion patterns not directly captured by the conventional initial work-up alone. We also clarified that DCR is intended to complement, rather than replace, the standard initial work-up and subsequent guideline-recommended testing. In practical terms, DCR can be performed in a general radiographic room using a flat-panel detector, a pulsed X-ray generator, and dedicated analysis software, which supports its potential role as a more accessible and immediate adjunct in the early diagnostic pathway.**

**Changes made in manuscript:**

**ORIGINAL**

**Page 5, Introduction**

**FIND-DCR is the first prospective comparative study to test, in a controlled multicenter setting, whether adding DCR-based pulmonary circulation analysis to the standard initial work-up improves the initial discrimination between CTEPH and non-CTEPH in patients with echocardiographically suspected PH.**

**REVISED**

**Page 5, Introduction**

**To date, there is no widely available tool for assessing pulmonary circulation at the time of initial clinical evaluation, particularly in non-expert or community settings. The standard initial work-up, including blood tests, chest radiography, ECG, pulmonary function tests, and echocardiography, provides solely indirect and often non-specific evidence of pulmonary hypertension and its potential causes. In contrast, DCR-based pulmonary circulation analysis provides additional dynamic regional perfusion-surrogate information derived from cardiac cycle-related changes in X-ray translucency during a short breath-hold, without the need for contrast media or radionuclides. FIND-DCR is the first prospective comparative study to test, in a controlled multicenter setting, whether adding DCR-based pulmonary circulation analysis to the standard initial work-up improves the initial discrimination between CTEPH and non-CTEPH in patients with echocardiographically suspected PH.**

Editor comment 2:

2. How does the study design control for reader-related biases, including inter-reader variability and potential learning or expectation effects when interpreting DCR-enhanced cases?
This question targets:

**Response:**

**We appreciate this important methodological point. We have expanded the Methods section to clarify how the protocol mitigates reader-related bias.**

**First, all case packages are de-identified, and readers are blinded to the reference-standard diagnosis, perfusion scintigraphy results, right heart catheterization findings, and subsequent clinical outcomes. Second, case-wise randomization is performed only after the reference-standard diagnosis has been established, and each case is interpreted only once in its allocated condition; readers do not review the same case both with and without DCR outputs. Third, all DCR readers undergo a prespecified training and qualification process before formal image interpretation. This approach is supported by prior work on DCR in CTEPH, in which readers were trained using representative CTEPH and non-CTEPH cases before image interpretation, and substantial inter-reader agreement for DCR was achieved (κ = 0.71, Ref. 1). Fourth, inter-reader variability is addressed in two ways in the present protocol: (i) the confirmatory primary analysis uses a prespecified adopted interpretation rule rather than a simple majority vote, and (ii) supportive analyses will evaluate the impact of reader-related factors, particularly years of clinical experience, on diagnostic accuracy in DCR interpretation. Taken together, these measures are intended to minimize bias related to repeated case review and inter-reader variability, while improving the reproducibility and methodological rigor of DCR interpretation.**

1. **Yamasaki Y, Abe K, Kamitani T, Hosokawa K, Hida T, Sagiyama K, et al. Efficacy of dynamic chest radiography for chronic thromboembolic pulmonary hypertension. Radiology. 2023;306(3):e220908.**

**Changes made in manuscript:**

**ORIGINAL**

**Page 15, Reader training, calibration, and image interpretation**

**During the formal reading phase, five DCR readers (internal medicine physicians) will interpret all randomized case packages independently in their allocated strategy using a standard case report form. For the primary endpoint, each reader will provide (i) a binary judgement (CTEPH vs non-CTEPH) and (ii) six-zone regional perfusion defect assessments. Non-evaluable interpretations due to non-diagnostic image quality are permitted and will be handled as prespecified.**

**REVISED**

**This training-based approach is supported by prior work on DCR in CTEPH, in which readers were trained using representative CTEPH and non-CTEPH cases before image interpretation and substantial inter-reader agreement for DCR was achieved (κ = 0.71) [7].**

**During the formal reading phase, five DCR readers will interpret all randomized case packages independently in their allocated strategy using a standardized case report form. Each case will be interpreted only once in its allocated condition, and readers will not review the same case both with and without DCR outputs. During the reading phase, no feedback regarding diagnostic correctness will be provided to the readers. For the primary endpoint, each reader will provide (i) a binary judgment (CTEPH vs non-CTEPH) and (ii) six-zone regional perfusion defect assessments. Non-evaluable interpretations due to non-diagnostic image quality are permitted and will be handled as prespecified.**

**ADDED**

**Page 17, Randomization, allocation, and blinding**

**To mitigate learning effects, formal readings will be performed independently, in randomized case order, using standardized case report forms, and without feedback regarding diagnostic correctness during the reading phase.**

**ADDED**

**Page 21, Statistical analysis**

**As a supportive analysis, multi-reader multi-case modelling will also be performed using all individual reads, treating reader and case as random effects [12]. Additional supportive analyses will evaluate the influence of reader-related factors, particularly years of clinical experience, on diagnostic accuracy in DCR interpretation.**

Editor comment 3:

3. How will discordant findings between DCR and V/Q scintigraphy be analyzed, particularly in cases where DCR identifies perfusion defects not seen on V/Q or vice versa

Response:

We thank the Editor for this important question. In this study, the reference standard is the site-level final diagnosis of CTEPH versus non-CTEPH, established by the treating investigators on the basis of the overall clinically indicated diagnostic work-up in accordance with guideline-recommended diagnostic criteria. This final diagnosis is based on multiple sources of information, generally including right heart catheterization, lung V/Q scintigraphy, echocardiography, biomarkers, and CT pulmonary angiography and/or catheter-based pulmonary angiography, as appropriate; in this study, right heart catheterization and lung V/Q scintigraphy are mandatory.

Within the blinded core laboratory, two complementary performance evaluations are planned. First, the primary performance evaluation compares the diagnostic performance of initial discrimination based on standard initial work-up alone with that based on standard initial work-up plus DCR-based pulmonary circulation analysis, using the site-level final diagnosis as the reference standard. This evaluation reflects the intended clinical positioning of the DCR-based pulmonary circulation analysis program and was defined in agreement with the PMDA. Second, as a secondary academic performance evaluation consistent with STARD 2015, the diagnostic performance of pulmonary blood flow defect detection by DCR-based pulmonary circulation analysis and by lung perfusion scintigraphy will be assessed in relation to the site-level final diagnosis of CTEPH versus non-CTEPH.

Accordingly, discordant findings between DCR and lung perfusion scintigraphy, such as DCR-positive/scintigraphy-negative or DCR-negative/scintigraphy-positive patterns, will not by themselves determine the primary endpoint, because the primary endpoint is defined against the final clinical diagnosis. Rather, such discordant findings will be interpreted within the secondary academic performance evaluation in relation to the site-level final diagnosis.

**We have revised the manuscript to clarify this analytical framework.**

**Changes made in manuscript:**

**Page 17, Reference standard and clinical verification**

**ADDED**

**The site-level final diagnosis of CTEPH versus non-CTEPH serves as the reference standard for the planned performance evaluations in the blinded core laboratory.**

**Page 18, Outcomes**

**ORIGINAL**

**STARD-based performance metrics (sensitivity, specificity, accuracy, positive predictive value, and negative predictive value) for DCR using V/Q scintigraphy as the reference standard (full-analysis set)**

**REVISED**

**STARD-based academic performance evaluation of pulmonary blood flow defect detection by DCR-based pulmonary circulation analysis and lung perfusion scintigraphy in relation to the site-level final diagnosis of CTEPH versus non-CTEPH (full-analysis set).**

--------------------------------------------------

Response to Reviewer #1

--------------------------------------------------

Major point 1:

1. A key question is how well-established Dynamic chest radiography (DCR) is in Japan. Is it widely available?

**Response:**

**We thank the reviewer for this important point.**

**Dynamic digital radiography (DDR) has been commercially available since around 2020. As of March 2025, it had been installed in 128 institutions in Japan, 192 institutions in the United States, and 379 institutions worldwide. Although these numbers suggest that DDR is not yet as widely disseminated as conventional static radiography systems, its future potential for broader adoption should be considered. Because the replacement cycle for radiographic equipment is generally around 10 years, and because the installation cost of static digital radiography and DDR is not substantially different, demonstration of the clinical utility of DDR could lead to rapid replacement of static digital radiography by DDR over the next 5 years.**

**In particular, ventilation-perfusion (V/Q) scintigraphy remains the standard examination for evaluating pulmonary circulation. However, its broader use is limited by several factors, including the availability of medical isotopes, the cost of installing nuclear medicine equipment, and concerns regarding the long-term sustainability of reactor-based isotope supply. For these reasons, there is increasing interest, especially in emerging and developing countries, in simpler and lower-cost tools for pulmonary circulation assessment, such as DCR.**

**In addition, in the diagnosis of CTEPH, V/Q scintigraphy is performed in nearly all cases in North America and Japan, whereas in Europe it is reportedly not performed in approximately one-third of cases [1]. Taken together, these considerations suggest that dynamic chest radiography has the potential to become a globally relevant alternative to lung perfusion scintigraphy in the diagnostic pathway for CTEPH.**

**Reference**

1. **Guth S, D'Armini AM, Delcroix M, Nakayama K, Fadel E, Hoole SP, Jenkins DP, Kiely DG, Kim NH, Lang IM, Madani MM, Matsubara H, Ogawa A, Ota-Arakaki JS, Quarck R, Sadushi-Kolici R, Simonneau G, Wiedenroth CB, Yildizeli B, Mayer E, Pepke-Zaba J. Current strategies for managing chronic thromboembolic pulmonary hypertension: results of the worldwide prospective CTEPH Registry. ERJ Open Res. 2021;7(3):00850-2020.**

**Changes made in manuscript:**

**Page, 3 Introduction**

**ADDED**

**Although dynamic digital radiography is not yet as widely disseminated as conventional static radiography, its clinical availability has been gradually increasing since its commercial introduction. Because it can be implemented in a general radiographic imaging environment without contrast media, radionuclides, or dedicated nuclear medicine infrastructure, DCR may offer a practical advantage in settings where access to V/Q scintigraphy is limited. This issue is particularly relevant given the logistical and economic constraints associated with scintigraphy, including radiotracer availability, equipment costs, and the sustainability of isotope supply. If its clinical utility is established, DCR-based pulmonary circulation analysis could become a more accessible tool for early pulmonary circulation assessment in both expert and non-expert settings.**

Major point 2:

2. Acute symptomatic pulmonary embolism on imaging within 3 months prior to enrolment is an exclusion. However, in Europe a recent PE is oftentimes the first evidence of CTEPH. In fact, a majority of CTEPH diagnoses in Europe emerge from an acute PE presentation (acute-on-chronic).

**Response:**

**We appreciate this insightful comment and agree that acute-on-chronic presentations are clinically important.**

**According to the 2022 ESC/ERS Guidelines for the diagnosis and treatment of pulmonary hypertension and the 2019 ESC Guidelines for the diagnosis and management of acute pulmonary embolism developed in collaboration with the European Respiratory Society (ERS), the diagnosis of CTEPH is defined after at least 3 months of appropriate therapeutic anticoagulation. In principle, DCR-based pulmonary circulation analysis may also detect acute pulmonary embolism, and retrospective proof-of-concept data supporting this possibility are available (Ref. 1). However, because the present study was designed to support regulatory approval of a medical device, discussions with the PMDA emphasized the importance of restricting the target condition to a single disease entity in order to evaluate diagnostic performance clearly and to define the intended indication after approval. For this reason, patients with complex conditions, including symptomatic pulmonary embolism within 3 months before enrollment, are excluded from the present study.**

**We also recognize that this design limits the generalizability of our findings to acute pulmonary embolism and to CTEPH diagnosed earlier in real-world clinical practice. We have added this point to the Discussion.**

**Changes made in manuscript:**

**Page 24, Discussion**

**ADDED**

**Another limitation is that patients with symptomatic acute pulmonary embolism within 3 months before enrollment are excluded to allow clearer evaluation of diagnostic performance in a single target disease entity. In addition, because this study enrolls patients with echocardiographically suspected pulmonary hypertension, the results may not be fully generalizable to acute-on-chronic presentations or to patients with less advanced chronic thromboembolic disease, including those with relatively preserved right ventricular structure/function or a lower echocardiographic probability of pulmonary hypertension.**

Major point 3:

3. One disadvantage of a ‘high-probability of PH’ approach is that authors will be missing all CTEPH cases with normal RVs. Should be acknowledged.
**Response:**

**We agree and thank the reviewer for pointing this out. We have now acknowledged this explicitly in the Discussion. The present study is designed to evaluate DCR within the clinical pathway of patients with echocardiographically suspected PH who are undergoing definitive work-up for CTEPH versus non-CTEPH. Accordingly, patients with less advanced disease, including some patients with chronic thromboembolic disease and relatively preserved right ventricular structure/function or lower echocardiographic probability of PH, may be underrepresented. We have added this limitation to improve the manuscript’s clinical framing.**

**Changes made in manuscript:**

**Page 24, Discussion**

**ADDED**

**Another limitation is that patients with symptomatic acute pulmonary embolism within 3 months before enrollment are excluded to allow clearer evaluation of diagnostic performance in a single target disease entity. In addition, because this study enrolls patients with echocardiographically suspected pulmonary hypertension, the results may not be fully generalizable to acute-on-chronic presentations or to patients with less advanced chronic thromboembolic disease, including those with relatively preserved right ventricular structure/function or a lower echocardiographic probability of pulmonary hypertension.**

Major point 4:

4. I am unclear on whether chest CT is part of the ‘case package’ that is sent to the core lab. To leave out CTPA is a significant disadvantage for the patients and is not GL recommended. Modern CT delivers iodine maps and parenchymal imaging that is diagnostically relevant. Authors should describe precisely how CTPA participates in this study. If a reader receives a ‘case package’ the diagnosis may be evident without DCR. Please, explain.

**Response:**

**We appreciate this important comment and agree that the role of CTPA should be stated more clearly. CTPA is not included in the blinded reader case package. This is intentional because the purpose of the reader study is to evaluate the incremental diagnostic contribution of DCR when added to the standard initial work-up, rather than to compare DCR against a fully advanced imaging work-up. If advanced imaging performed later at expert or tertiary centers, such as CTPA (including iodine maps) or pulmonary angiography, were included in the case package, there would be little room for DCR-based pulmonary circulation images to contribute meaningfully to improving diagnostic accuracy for CTEPH.**

**The intended clinical position of DCR-based pulmonary circulation analysis is in the initial diagnostic phase. Its highly innovative value lies in enabling pulmonary circulation assessment in patients presenting with dyspnea in a simple, non-contrast, low-radiation manner, even at the level of regional hospitals or community clinics.**

Minor point 5:

5. PPS should be mentioned as an exclusion.

**Response:**

**We appreciate this comment. We had recognized peripheral pulmonary artery stenosis (PPAS) as a representative disease that can mimic CTEPH and therefore as a specific condition that should be excluded by design. The same consideration applies to other non-CTEPH Group 4 pulmonary hypertension conditions, such as pulmonary artery sarcoma and Takayasu arteritis, which may also mimic CTEPH and affect diagnostic performance.**

**For this reason, we included the exclusion criteria “congenital or acquired abnormalities of pulmonary vascular anatomy” and “history of vasculitis” in order to exclude these non-CTEPH pulmonary vascular disorders in a broader and practical manner. In actual study operation, inquiries regarding PPAS have also been managed consistently so that such cases are not included as eligible participants.**

Minor point 6:

6. The title of the study should be DCR-FIND, and not FIND-DCR.

**Response:**

**We appreciate the reviewer’s suggestion. However, we have respectfully retained the acronym FIND-DCR because this study name is already used in the clinical investigation plan, internal study documents, and trial registration. Retaining the registered acronym avoids inconsistency between the manuscript and the official trial record.**

Minor point 7:

7. Replace V/Q scintigraphy with perfusion scintigraphy because that is what you are planning to do

**Response:**

**We thank the reviewer for this helpful comment. In this protocol, ventilation–perfusion (V/Q) scintigraphy and lung perfusion scintigraphy are intentionally distinguished and used in different contexts. Specifically, in the secondary endpoints, the STARD 2015–based performance evaluation and the six-zone regional assessment compare lung perfusion scintigraphy with DCR-based pulmonary circulation images. In contrast, for the clinical final diagnosis, V/Q scintigraphy is required in accordance with current diagnostic guidelines for CTEPH.**

**We have revised the manuscript to make this distinction clearer and more consistent throughout the text.**

**We again thank the Editor and Reviewer for their valuable comments, which have substantially improved the clarity and rigor of our manuscript.**

Sincerely,

Kazuya Hosokawa, MD, PhD

Corresponding author
